# A Functional Yeast Survival Screen of Tumor-Derived cDNA Libraries Designed to Identify Anti-Apoptotic Mammalian Oncogenes

**DOI:** 10.1371/journal.pone.0064873

**Published:** 2013-05-22

**Authors:** Moritz Eißmann, Bettina Schwamb, Inga Maria Melzer, Julia Moser, Dagmar Siele, Ulrike Köhl, Ralf Joachim Rieker, David Lukas Wachter, Abbas Agaimy, Esther Herpel, Peter Baumgarten, Michel Mittelbronn, Stefanie Rakel, Donat Kögel, Stefanie Böhm, Tony Gutschner, Sven Diederichs, Martin Zörnig

**Affiliations:** 1 Chemotherapeutisches Forschungsinstitut Georg-Speyer-Haus, Frankfurt/Main, Germany; 2 Institute of Cellular Therapeutics, IFB-Tx, Hannover Medical School, Hannover, Germany; 3 Institute for Pathology, University Hospital Erlangen, Erlangen, Germany; 4 Institute of Pathology, University of Heidelberg, Heidelberg, Germany; 5 Institute of Neurology (Edinger Institute), Frankfurt/Main, Germany; 6 Experimental Neurosurgery, Center for Neurology and Neurosurgery, Goethe University Hospital Frankfurt, Frankfurt/Main, Germany; 7 Helmholtz-University-Group Molecular RNA Biology & Cancer, German Cancer Research Center (DKFZ), Heidelberg, Germany; University of Texas Health Science Center at San Antonio, United States of America

## Abstract

Yeast cells can be killed upon expression of pro-apoptotic mammalian proteins. We have established a functional yeast survival screen that was used to isolate novel human anti-apoptotic genes overexpressed in treatment-resistant tumors. The screening of three different cDNA libraries prepared from metastatic melanoma, glioblastomas and leukemic blasts allowed for the identification of many yeast cell death-repressing cDNAs, including 28% of genes that are already known to inhibit apoptosis, 35% of genes upregulated in at least one tumor entity and 16% of genes described as both anti-apoptotic in function and upregulated in tumors. These results confirm the great potential of this screening tool to identify novel anti-apoptotic and tumor-relevant molecules. Three of the isolated candidate genes were further analyzed regarding their anti-apoptotic function in cell culture and their potential as a therapeutic target for molecular therapy. PAICS, an enzyme required for *de novo* purine biosynthesis, the long non-coding RNA *MALAT1* and the MAST2 kinase are overexpressed in certain tumor entities and capable of suppressing apoptosis in human cells. Using a subcutaneous xenograft mouse model, we also demonstrated that glioblastoma tumor growth requires MAST2 expression. An additional advantage of the yeast survival screen is its universal applicability. By using various inducible pro-apoptotic killer proteins and screening the appropriate cDNA library prepared from normal or pathologic tissue of interest, the survival screen can be used to identify apoptosis inhibitors in many different systems.

## Introduction

Apoptosis is a common form of programmed cell death occurring in metazoans that leads to removal of cells in the organism while avoiding the induction of inflammation [1], [2]. Two distinct but interconnected apoptotic signaling pathways have been discovered and delineated at the molecular level. The extrinsic pathway is physiologically triggered by ligands of the death receptor family, which leads to receptor clustering, assembly of the cytoplasmic receptor complex DISC (death inducing signaling complex) and activation of initiator CASPASE-8 and CASPASE-10 within the DISC platform [3]. The intrinsic pathway involves the death stimulus-induced release of mitochondrial Cytochrome c (CYT c) into the cytoplasm, where it triggers multimerization of the adaptor protein, APAF-1, and formation of the apoptosome complex, which ultimately enables CASPASE-9 recruitment and activation [4].

While apoptosis functions to coordinate the elimination of excess, hazardous or damaged cells under normal physiological conditions [2], [5], alterations in the regulatory mechanisms of cell death/survival contribute to many human pathological conditions, including cancer and neurodegenerative diseases, thus highlighting the importance of maintaining the tight regulation of the apoptotic machinery [6]. From the onset of the transformation process, cancer cells are exposed to a variety of strong apoptotic stimuli, such as oncogene activation, hypoxia and anoikis [7]–[10]. Therefore, potent selective pressure may cause unphysiological activation of survival signals (such as gain-of-function mutations, overexpression of anti-apoptotic proteins) and accumulation of loss-of-function mutations (i.e. inactivation of pro-apoptotic tumor suppressors). Nevertheless, tumor cells are usually more sensitive to external apoptotic stimuli compared with their non-transformed counterparts, thereby indicating that despite acquisition of apoptosis-inhibiting mutations, they are pushed to the edge of survival by environmental (and proliferative) stresses [11], [12]. As a consequence, first-line chemotherapy and radiation normally eliminate the primary tumor mass before remaining tumor (stem) cells acquire additional anti-apoptotic mutations, which render the cells insensitive to therapeutic intervention [13]. The developing resistant tumor metastases represent a severe clinical problem and significantly contribute to the cancer-related death rate [14].

The identification of all relevant anti-apoptotic oncoproteins and their evaluation for targeted molecular therapy, in combination with other treatment options, is required to enhance therapeutic treatments and alternatives. Therefore, we have developed a functional fission yeast-based survival screen of cDNA libraries prepared from human tumors to identify novel anti-apoptotic oncoproteins (over-) expressed in various human tumor entities. Yeast cells can be efficiently killed by heterologous overexpression of several pro-apoptotic molecules, including pro-apoptotic members of the BCL-2 protein family and adaptor proteins such as CED-4/APAF-1 [15]–[17]. The specificity of the process is highlighted by the observation that mutants of the pro-apoptotic molecules that are incapable of killing mammalian cells do not induce yeast cell death [15]. In addition, the demise of yeast cells can be inhibited by co-expression of the appropriate anti-apoptotic proteins. For example, the co-expression of the mammalian apoptosis inhibitor, BCL-X_L_, and the pro-apoptotic BCL-2 family member, BAK, prevents BAK-induced yeast cell death [15]. Based on this finding, the unicellular yeast model can be used as a high throughput screening system to identify inhibitory molecules that interfere with the yeast cell death program triggered by a killer protein downstream of the stimulus or by directly binding and neutralizing the pro-apoptotic protein [18]–[20]. Preliminary small-scale screen results have indicated that a majority of the yeast cell death-suppressing cDNAs are also capable of inhibiting apoptosis in mammalian cells, thereby validating the potential of the screening system [21].

We prepared high quality mRNA from primary human tumor material of various origins, including a lung metastasis of a melanoma, glioblastoma and therapy-resistant leukemic blasts. By selecting tumor entities and metastases that are notoriously resistant to chemotherapy-induced apoptosis [22], [23], we increased the chances of isolating anti-apoptotic proteins that are important for tumor initiation and the development of therapy resistance. Such tumor cell death inhibitors may represent attractive novel targets for molecular therapeutic strategies.

Here, we present the principle and representative results of the yeast survival screenings, which includes statistics and experimental cell culture and *in vivo* data for the isolated anti-apoptotic candidate oncogenes, namely *PAICS*, *MALAT1* and *MAST2*. The majority of the library cDNAs isolated from the surviving yeast colonies interfered with mammalian apoptosis. By demonstrating the oncogenic potential of the identified genes in human cells both in cell culture and *in vivo*, we confirmed the power of this functional yeast screening system, which can be broadly applied and adapted to isolate relevant apoptosis inhibitors in all biological and patho-physiological metazoan systems in which regulation and de-regulation of apoptosis is very important, such as during development [24], [25] and autoimmunity [26].

## Materials and Methods

### Ethics statement

Approval of the mouse experiments was obtained prior to beginning of the research by the Hessian Regional Commission in Darmstadt, Germany (reference number V54-19c20/15-F123/33). The procedure of injecting genetically modified human cancer cell lines into the flanks of NOD/SCID mice and killing the animals for analytical purposes in the laboratory of Dr. Martin Zörnig (Georg-Speyer-Haus, Frankfurt, Germany) was approved by the animal welfare officer, Dr. Margit Wagenblast.

For the mouse experiments described in this manuscript, 100 µl of tumor cell suspension were injected once subcutaneously into the flank of the animals, which had been anesthetized before by inhalation of isoflurane. Tumors were measured 2–3 times per week, and when the tumor volume reached 1 cm^3^, the animals were euthanized by sufficient inhalation of isoflurane, followed by cervical dislocation and removal of the tumor mass for further analysis.

### Yeast and mammalian cell lines

Growth conditions and media for the fission yeast *Schizosaccharomyces pombe* were described previously [27]. The *S.pombe* yeast strain DSI was established upon stable transfection of the yeast expression construct *pRIP45-hBAK* [15] and HC4 by stable transfection of *pRIP45-CED-4* [16]. In the *RIP45* vector, thiamine-repressable transgene expression is under control of the *nmt*-promotor [28], [29].

The melanoma cell line MelJuSo (DSMZ (German collection of microorganisms and cells cultures) no. ACC 74) and the glioblastoma cell line U87 (ATCC no. HTB-14) were used for cell culture and *in vivo* tumor xenograft experiments. The lung carcinoma cell line A549 (ATCC no. CCL-185) was used to establish zinc finger nuclease (ZFN)-mediated *MALAT1* knockout cell clones (A549 *MALAT1* ko1 and ko2) [30]. Parental A549 cells and A549-ctr (control) cell clones (with the *GFP*-polyA expression cassette randomly integrated; A549-ctr clones exhibited unaffected *MALAT1* levels similar to the parental cells) were used as controls.

### Reagents and media

Doxycycline, epoxomicin, lithium acetate (LiOAc), MG132, phloxine B, polyethylene glycol (PEG; P4000) and thiamine were purchased from Sigma-Aldrich (Taufkirch, Germany). Recombinant TRAIL was obtained from PeproTech Inc. (Rocky Hill, USA), and Edinburgh Minimal Medium (EMM), L-leucine, uracil and yeast extract sucrose (YES) were purchased from MP Biochemicals (Heidelberg, Germany). Dulbecco's modified eagles medium (DMEM) and Roswell Park Memorial Institute (RPMI) medium (both Invitrogen, Karlsruhe, Germany) were used for mammalian cell culture.

### Tumor-derived yeast expression cDNA libraries

Primary tumor material (a lung metastasis from a primary melanoma, biopsies from 6 different glioblastomas, and treatment-resistant leukemic blasts isolated from 6 patients) was used to isolate high-quality polyA^+^ mRNA, which was then reversely transcribed into full length cDNA, employing the SMART-method [31]. The cDNA fragments were cloned by directed insertion into the constitutively active *S. pombe* expression vector *pART1b* [21]. The primers *pART1b LCK fw* (5′-ATCCTTTCGCAAAAACTCGGT-3′) and *pART1b rev* (5′-TGTAAATCATCTGATGGAGGA-3′) were used for sequencing of cDNA inserts.

### Functional yeast survival screen

With single DSI [15] and HC4 [16] yeast colonies, for which cell death following killer protein induction had been confirmed, a 5 ml pre-culture containing 5 µg/ml thiamine was inoculated overnight while shaking at 30°C. Next, 4 ml of this culture was added to 150 ml thiamine-containing medium, and following overnight growth, 100 ml of this stationary phase yeast culture was mixed with 1 l EMM yeast medium containing either leucine, uracil and thiamine (DSI) or leucine and uracil without thiamine (HC4) until they reached an OD_595_ density of 0.3. Yeast cells were grown for three hours until they had reached an OD_595_ of 0.6 and then harvested by centrifugation at 1,000 g at room temperature for 5 min. The cell pellet was washed with 800 ml water, transferred to 15 ml Falcon tubes and centrifuged again. The resulting yeast pellets were resuspended in 8 ml LiOAc/1xTE and then transferred to a Falcon tube containing 20 mg denatured herring sperm carrier-DNA and 150 µl library cDNA (∼100 µg). For transformation of the HC4 strain, 30 µl of library cDNA (20 µg) were used. Next, 60 ml PEG/LiOAc were added to the yeast/DNA mixture, and the solution was then divided into two 50 ml Falcon tubes and incubated for 30 min at 30°C, shaking at 150 rpm. 10% DMSO (7 ml) was added and yeast were incubated in a 42°C water bath for 15 min “heat-shock” with frequent shaking. Afterwards, yeast cells were kept on ice for 3 min and then centrifuged for 5 min at 1,000 g at room temperature. The pellets were resuspended in 6 ml 1xTE buffer and the yeast cell suspension was distributed and plated on 14.5 cm yeast agar plates with EMM agar plus uracil and thiamine. For the HC4 screen, the suspension was plated directly on EMM plates plus uracil (without thiamine). To estimate the transformation efficiency, 20 to 50 µl transformed yeast cell aliquots (40–200 ng cDNA) were diluted with 1xTE and then plated on 10 cm yeast agar plates with EMM agar plus uracil and thiamine. After four to six days, yeast colonies from the test plates were counted and transformation efficiencies were determined. The 14.5 cm screening plates were incubated for four to six days at 30°C. After apparent colony formation, the plates were replica plated up to four times on thiamin-free agar plates with EMM agar plus uracil but without thiamine for the selection of surviving colonies. Agar plates containing phloxine (5 µg/ml) were used to allow for better discrimination between pale living colonies (phloxine-free) and red dead yeast colonies [32].

### shRNA constructs for cellular knockdown experiments

*pGIPZ* shRNA vectors were purchased from Open Biosystems (Lafayette, USA; for further information see www.openbiosystems.com). The non-silencing control vector *pGIPZ shctr* and the following three *PAICS*-specific *pGIPZ* shRNAs were used for non-inducible knockdown experiments:

*pGIPZ shctr*: hairpin 5′- TGCTGTTGACAGTGAGCGATCTCGCTTGGGCGAGAGTAAGTAGTGAAGCCACAGATGTACTTACTCTCGCCCAAGCGAGAGTGCCTACTGCCTCGGA -3′ (sense and anti-sense sequence are underlined)

*pGIPZ sh1PAICS*: hairpin 5′-TGCTGTTGACAGTGAGCGATTTTCTAGCTGCATTTCCTTAGTGAAGCCACA GATGTAAGGAAATGCAGCTAGAAAAGTGCCTACTGCCTCGGA-3′

*pGIPZ sh2PAICS*: hairpin 5′- TGCTGTTGACAGTGAGCGCGGGCTCCAAATGGTAAAGAAATAGTGAAGCCACAGATGTATTTCTTTACCATTTGGAGCCCTTGCCTACTGCCTCGGA -3′

*pGIPZ sh3PAICS*: hairpin 5′-TGCTGTTGACAGTGAGCGATCTTAATCTATAAACCATGTAGTGAAGCCACA GATGTACATGGTTTATAGATTAAGAGTGCCTACTGCCTCGGA -3′

To obtain doxycycline-inducible *pTRIPZ* sh*ctr* and *pTRIPZ* sh2*PAICS* constructs, the short hairpins from the corresponding *pGIPZ* vectors were subcloned into *pTRIPZ* following the *Open Biosystems* manual.

*pLKO.1-puro* vectors from Sigma-Aldrich (Taufkirch, Germany) were used to downregulate *MAST2* expression (sh1*MAST2* RNA: 5′- CCGGTGTGGACATGGTGCGTCTATACTCGAGTATAGACGCACCATGTCCACATTTTTG -3′ (sense and anti-sense underlined); sh2*MAST2* RNA: 5′-CCGGGAGGACTTCGAGACCATTAAGCTCGAGCTTAATGGTCTCGAAGTCCTCTTTTTTG -3′; non-silencing control shRNA (sh*ctr*): 5′- CCGGCAACAAGATGAAGAGCACCAACTCGAGTTGGTGCTCTTCATCTTGTTGTTTTTG -3′.

### Lentiviral transduction of mammalian cells

HEK 293T cells were seeded (2×10^6^ cells per 10 cm dish) and transfected with 5 µg of total DNA (2.5 µg lentiviral construct plus 1.625 µg packaging plasmid *p8.91* and 0.875 µg packaging plasmid *pMD2.G*, which both encode structural virus proteins) using the polyethyleneimine method. Virus-containing supernatant was harvested 48 and 72 hours after transfection and filtered through a 0.45 µg syringe filter unit. The virus was then either stored at 4°C for up to one week or, following concentration by ultracentrifugation, at −80°C for long-term storage. To concentrate the virus, virus-containing supernatant (max. 30 ml) was transferred into open-top thinwall polyallomer tubes, and 5 ml 20% sucrose was carefully pipetted into the bottom of each tube as underlayer. Tubes were then inserted into the pre-cooled centrifuge rotor buckets and tared. The viral supernatant was centrifuged for 2 h 20 min at 4°C and 20,500 rpm (51,610 g). Next, the supernatant was decanted and the tubes were turned upside down on a tissue for a few seconds to drain the remaining liquid. The virus particles were dissolved in 200 µl −1 ml of the desired culture medium for at least 2 h (or overnight) at 4°C. The concentrated virus was stored at −80°C.

For the transduction of adherent cells, 2×10^5^ cells per well (6-well plates) were seeded 24 hours before transduction. The lentiviral supernatant or concentrated virus was mixed with fresh medium, polybrene was added with a final concentration of 8 µg/ml and cells were then spin-infected by centrifugation at 250 g and 32°C for 60 min. After an additional 4 to 6 hours of incubation at 37°C and 5% CO_2_, the virus-containing medium was removed and fresh culture medium was added. When the cells had grown to confluency, they were trypsinized and transferred to 10 cm dishes. This is referred to as passage zero (P0) *post transduction*.

### cDNA synthesis using the Omniscript RT kit

The Omniscript Reverse Transcriptase kit (Qiagen) was used for reverse transcription of RNA species according to the manufacturer's instructions. Briefly, 1 µg of RNA was transcribed in a 20 µl reaction mix containing RT reaction buffer, dNTPs, RNAse inhibitor (RiboLock, Fermentas), reverse transcriptase, oligo(dT) and random hexamer primers. The reaction mixture was incubated for 1 h at 37°C.

### cDNA synthesis (alternative method)

Long RNA molecules, such as *MALAT1*, can form secondary structures that reduce the efficiency of these molecules as templates in the reverse transcription reaction. To prevent secondary structure formation, the RNA is pre-heated prior to cDNA synthesis and reverse transcription is performed at higher temperatures with special enzymes optimized for the synthesis of long cDNAs, such as the RevertAid H Minus Reverse Transcriptase (Fermentas). Next, 1 µg RNA was mixed with 2 µl random hexamer primers (100 µM) and 12 µl DEPC-H_2_O, heated for 5 min at 70°C, cooled on ice and then centrifuged for 3 sec at 1,800 rpm. A reaction mixture containing 4 µl 5× RT-buffer, 2 µl dNTP mix (10 mM of each dNTP), 1 µl Ribolock RNase inhibitor (40 U/µl) and 1 µl RevertAid H minus Reverse Transcriptase (200 U/µl) was added to the RNA sample and incubated at 42°C for 1 h. After an additional 10 min incubation at 70°C, the reaction mixture was transferred to ice and used for Real-Time PCR analysis.

### Quantitative Real-Time PCR (qPCR)

The relative quantification of mRNA transcripts was performed using the SybrGreen method [33]. The specificity of the qPCR reaction was verified by assessing the melting curves, via agarose gel analysis, and sequencing of the isolated reaction product. cDNA was prepared using either the Omniscript RT kit (Qiagen) or the above-described alternative protocol. qPCR analysis was performed using an iCycler iQ (*Biorad*). Individual samples were analyzed in triplicate. To compare the relative abundance of target cDNA in different samples, the ΔΔC_t_ value [34] was calculated as ΔΔC_t_  =  ΔC_t_ (sample 1)–ΔC_t_ (sample 2). The fold difference between each target gene compared with the housekeeping gene was calculated as 2^−ΔΔCt^.

### qPCR primer

Knockdown efficiencies were confirmed by quantitative real-time PCR (see Supplementary material and methods) using the following primers:

*MALAT1* (fw 5′- GAATTGCGTCATTTAAAGCCTAGTT -3′, rev 5′- GTTTCATCCTACCACTCCCAATTAAT -3′)

*RN7SL1* (for normalization; fw: 5′-ATCGGGTGTCCGCACTAAGTT-3′, rev: 5′-CAGCACGGGAGTTTTGACCT-3′)

*MAST2* (fw 5′- GGACAAGTAACCGCAAGAGC -3′, rev 5′-GAGCAGGTGCATTTGGAGAG-3′)

*HGPRT* (for normalization; fw 5′-TGACACTGGCAAAACAATGCA-3′, rev 5′-GGTCCTTTTCACCAGCAAGCT-3′)

### Super-SAGE expression analysis

Super-SAGE data for *MALAT1* expression in pancreas carcinoma and pancreatitis compared to normal pancreas tissue were obtained from *GenXPro GmbH* (Frankfurt am Main, Germany; Dr. Björn Rotter) and PD Dr. Christoph Michalski (Chirurgische Klinik und Poliklinik, Technische Universität München, Germany).

### Western blot analysis

Proteins separated by electrophoresis were transferred onto 0.45 µm nitrocellulose membranes using a semi-dry transfer system. For immunodetection, the unspecific binding of antibodies to the nitrocellulose membrane was inhibited via membrane incubation in blocking buffer for 1 h at room temperature or at 4°C overnight. Membranes were incubated with antiserum at 4°C overnight and washed three times for 10 min with PBS-T (0.1% Tween in 1× PBS). Secondary antibodies coupled to horseradish peroxidase were used for membrane incubation for 1 h at room temperature. After washing the membranes three times for 10 min with PBS-T, chemiluminescence was assessed using an ECL detection kit.

### Antisera

Induced expression of human BAK and *C. elegans* CED-4 proteins was confirmed using a polyclonal rabbit anti-BAK antibody from Santa Cruz (clone H211; sc-7873) and a rabbit anti-CED-4 antiserum (no. 9104.1, kindly provided by Dr. Gartner, Wellcome Trust Centre for Gene Regulation and Expression, University of Dundee, UK; [35]. For detection of the PAICS protein, immunohistochemical and Western blot analyses were performed with a self-raised anti-PAICS antiserum. The serum was retrieved from rabbits immunized with peptide A (GKKLYEGKTKEVYE; PAICS aa 10–23) and peptide B (RLWPSGDRSQQKDK; aa 222–235) and was affinity-purified using both peptides coupled to a column. For Western blot analysis, equal protein loading was confirmed with an anti-EZRIN antiserum (clone 3C12; Cat. No 35-7300; *Invitrogen*), or by Ponceau S staining of the membrane following protein transfer.

### Immunohistochemical analysis

A tissue microarray (TMA) was assembled by obtaining 2–3 tissue cores from conventional paraffin blocks of metastatic cutaneous malignant melanoma (total: 39 tumors), in addition to 25 cores from normal skin tissue containing epidermal melanocytes and epithelium. The cores were embedded into a recipient TMA block, from which 2 μ-sections were cut, mounted on superfrost glass slides, deparaffinized with xylene and rehydrated with graded ethanol. A multi-tumor array and a multi-(normal) tissue array were obtained from the National Center for Tumor Diseases (NCT Heidelberg, Germany), and the array sections were treated similar to the melanoma array sections. Slides were subjected to 5 min heating at 120° C in Tris-EDTA-Buffer pH 8.5 in a pressure cooker. Endogenous peroxidase activity was quenched by a 5 min incubation with Peroxidase-Blocking solution (catalog no. s2023, Dako Hamburg, Germany) at room temperature and afterwards, slides were washed with a wash buffer for 5 min (catalog no. 3006, Dako Hamburg, Germany). Afterwards, the sections were incubated with a with a self-raised polyclonal rabbit anti-PAICS antiserum (dilution 1∶50) for 30 min and washed for 5 min with a wash buffer (catalog no. 3006, Dako Hamburg, Germany). Slides were subsequently incubated with a labelled polymer (Envision, catalog no. K5007, Dako Hamburg, Germany) for 30 min and washed for 5 min (catalog no. 3006, Dako Hamburg, Germany). Antibody binding was visualized using 3,3′-diaminobenzidine in chromogen solution (DAB+ Substrate, catalog no. K 5007, Dako Hamburg, Germany). Tissue sections were counterstained with hematoxylin (catalog no. 1051750500, Merck, Darmstadt, Germany).

For MAST2 immunohistochemistry, human cortex tissue and U87 glioma cells were formalin-fixed and paraffin-embedded. The use of human tissue was approved by the ethics committee at the university hospital of Frankfurt. Paraffin blocks were cut with a microtome (3 µm thickness) and placed on SuperFrost-Plus slides (Microm International, Walldorf, Germany). A rabbit polyclonal anti-human MAST2 antibody (dilution 1∶50; catalog no. HPA040155; Sigma, Stockholm, Sweden) was used for immunohistochemistry. Tissue labelling was performed using the DiscoveryXT immunohistochemistry system (Ventana/Roche, Strasbourg, France).

### Apoptosis assay

The Nicoletti protocol was used for simultaneous quantification of apoptosis and cell cycle analysis [36]. First, cells were harvested and transferred to FACS tubes. After centrifugation (5 min, 400 g, 4°C), the cells were washed with PBS and again spun down by centrifugation. Cells were fixed by adding 1 ml ice-cold 70% ethanol dropwise while vortexing and then incubated at 4°C for at least 12 h. Before staining, cells were washed with 38 mM Na-Citrate (pH 7.4), centrifuged, and 200–400 µl of 38 mM Na-Citrate containing 50 µg/ml propidium iodide and 5 µg/ml RNase A was added. Cell staining was performed for 20 min at room temperature in the dark, and the acquisition of the cell cycle profiles in the FL2-A channel was performed with a FACS Calibur (Becton Dickinson Biosciences) using the doublet discrimination module (DDM) of the CellQuest Pro software (Becton Dickinson Biosciences).

### Colony formation assays

#### Methyl cellulose colony forming assay

MelJuSo melanoma cells lentivirally tranduced with either *pTRIPZ* sh*PAICS* or *pTRIPZ* sh*ctr* were treated for 3 days with 1 µg/ml doxycycline. Next, 16 hours before seeding for the assay, the cells were either incubated with 0.2 µM staurosporine or left untreated. Duplicates of 1×10^3^ MelJuSo cells were then seeded in 35 mm^2^ dishes (Nunc) in MethoCult® H4100 (Stemcell Technologies) in RPMI medium supplemented with 30% FBS, 3% L-glutamine, 3% penicillin/streptomycin and 3 µg/ml doxycycline. After 6 days of growth, red fluorescent colonies were quantified using a microscope.

#### Crystal violet colony forming assay

The A549 control GFP cells and two zinc finger nuclease (ZFN)-mediated *MALAT1* knockout cell clones were seeded in DMEM/10% FCS medium at 750 cells/well in triplicates in a 6-well plate. The colonies were allowed to grow for 8 days and were then washed once with PBS prior to staining and fixing with crystal violet solution (0.5% crystal violet and 6% glutaraldehyde) for 20 minutes. The plates were washed twice in distilled water and then dried. Colonies comprised of 60 cells and more were counted using a 3×3 cm scoring grid.

### Quantification of CASPASE-3-like protease activity

For measuring effector caspase activity, cells were lysed in 200 µl lysis buffer [10 mM HEPES, pH 7.4, 42 mM KCl, 5 mM MgCl_2_, 1 mM phenylmethylsulfonyl fluoride (PMSF), 0.1 mM EDTA, 0.1 mM EGTA, 1 mM dithiothreitol (DTT), 1 µg/ml Pepstatin A, 1 µg/ml Leupeptin, 5 µg/ml Aprotinin, 0.5% 3-(3-cholamidopropyldimethylammonio)-1-propane sulfonate (CHAPS)]. Fifty µl of this lysate were added to 150 µl reaction buffer (25 mM HEPES pH 7.5, 1 mM EDTA, 0.1% CHAPS, 10% sucrose, 3 mM DTT). The fluorogenic substrate Ac-DEVD-AMC was added at a final concentration of 10 µM. Accumulation of AMC fluorescence was monitored over two hours using an HTS fluorescent plate reader (excitation 380 nm, emission 465 nm). Protein content was quantified using the *Roti®-Quant* Coomassie Plus Protein Assay reagent (Roth, Karlsruhe, Germany). The caspase activity is expressed as a change in fluorescence units per µg protein and hour.

### Cell expansion assay

The total cell number and the number of viable cells in a sample were determined using a CASY® Cell Counter (Schärfe Systems, Reutlingen, Germany). The appropriate measurement program was established using Casyblue according to the manufacturer's instructions. For measurement, 25 µl cell suspension aliquots were transferred to a CASY® cup containing 10 ml CASY®ton, mixed by inverting three times and placed in the CASY® Cell Counter.

### Click-iT® EdU cell proliferation assay

S-phase analysis was performed with a *BD FACS Calibur* using the Click-iT® EdU Flow Cytometry Assay Kit (Alexa 488; Molecular Probes; #C35002) according to the manufacturer's instructions.

### Xenograft tumor mouse model

NOD-SCID mice (6 to 10 weeks old) were used for xenograft transplantation experiments. Three days before transplantation, mice were shaved on the back (right flank). Mice were anesthetized by isoflurane inhalation, and 5×10^6^ human cancer cells (in 100 µl PBS) were injected subcutaneously into the right flank. For the MelJuSo *pTRIPZ* PAICS shRNA and *pTRIPZ ctr* shRNA cells, mice received drinking water containing 2 mg/ml doxycycline and 10 g/l sucrose *ad libitum* until the end of the experiment to induce the *PAICS* shRNA-mediated knockdown. For the subcutaneous U87 xenograft model, a 100 µl cell suspension, containing 5×10^6^ U87 cells with or without MAST2 knockdown in 20 µl matrigel (BD Bioscience) and 80 µl PBS, was injected into each animal. Matrigel suspensions were kept on ice to avoid clogging of the injecting needle. Tumor growth was measured 2–3 times per week with a manual caliper. Mice were sacrificed when they developed ulcerating tumors or developed a xenograft tumor larger than 1 cm^3^. The tumor size was calculated as follows: tumor volume [mm^3^]  =  length × width^2^ ×0.5.

### Statistics

All statistical analyses were performed with GraphPad Prism 5.0 from GraphPad Software Inc. (La Jolla, USA). Data were presented as mean ± standard error of the mean (SEM) if not indicated otherwise. Two-tailed student's *t*-test (two group comparisons) or one-way-ANOVA analysis (multiple group comparisons) were performed, and statistical significance was indicated with * for p<0.05, ** for p<0.01 or *** for p<0.001.

## Results

### Screening of human tumor-derived cDNA expression libraries in BAK- and CED-4-expressing *S.pombe* yeast strains

**Figure 1:**
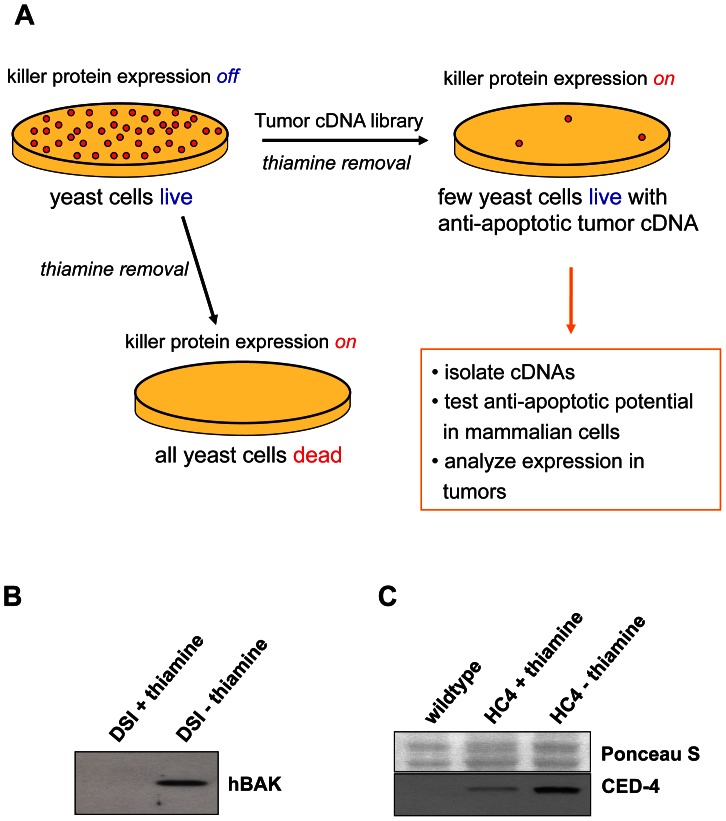
Principle of the yeast survival screen. **A**. Two *S. pombe* yeast strains were established with inducible expression of the pro-apoptotic proteins BAK and CED-4, following thiamine removal from the growth medium. Killer protein expression resulted in efficient yeast cell death upon plating onto thiamine-deficient yeast agar plates. Transformation of the yeast cells with a tumor-derived cDNA library led to survival of few killer protein-expressing yeast colonies from which the yeast cell death-inhibiting library cDNA insert was identified and analyzed for its anti-apoptotic potential in mammalian cells and expression levels in tumor biopsies. **B**,**C**. Inducible expression of human BAK (**B**) and *C. elegans* CED-4 (**C**) in yeast *S. pombe*. Total cell lysates were prepared from the parental yeast (wildtype), DSI- and HC4-strains cultured in thiamine-containing medium or after cultivation for 20 hours in thiamine-free medium. Subsequently, Western blot analysis was performed. Human BAK was detected with a polyclonal rabbit anti-BAK antibody (*Santa Cruz*, Heidelberg, Germany). Anti-CED-4 antiserum (9104.1, kindly provided by A. Gartner, University of Dundee, UK) was used at a 1∶500 dilution to detect *C.elegans* CED-4 expression. Ponceau S staining is presented as a protein loading control.

We used *S. pombe* fission yeast in a high throughput screening system to identify novel human anti-apoptotic oncoproteins. The principle of the screen, which is outlined in **Fig. 1A**, is based on the observation that the unicellular yeast organism can be killed upon heterologous (over-) expression of pro-apoptotic mammalian proteins [15]. To achieve inducible expression of death-inducing pro-apoptotic “killer” proteins in yeast cells, we cloned the human pro-apoptotic BCL-2 family member, *BAK*, into the thiamine-repressible *S. pombe* expression vector *pRIP45* and stably integrated the construct into the *S. pombe* genome. The resulting yeast strain, DSI (BAK), displayed significant killer protein expression following thiamine removal (see **Fig. 1B**) and died with great efficiency after three rounds of replica plating on thiamine-free yeast agar plates. In previous experiments, we had demonstrated quantitative cell death in the DSI strain in liquid culture 24 hours after *BAK* induction [15].

Additionally, we used another *S. pombe* yeast strain with inducible killer protein expression, HC4, in which the *C. elegans* homologue of *APAF-1*, *CED-4* [37], had been cloned into the *pRIP45* vector and stably integrated. The HC4 cells express elevated levels of CED-4 upon thiamine removal (**Fig. 1C**), thereby leading to rapid and complete yeast cell death as quantified in re-plating experiments [38]. The HC4 yeast strain was originally used to functionally investigate the *C. elegans* cell death machinery [16]. APAF-1 and CED-4 are structurally and functionally conserved, and ectopic CED-4 expression induces mammalian cell death which is inhibited by BCL-x_L_ overexpression [39]. Therefore, we used HC4 cells to screen for inhibitors of mammalian mitochondrial apoptosis. We have previously identified the APAF-1-binding anti-apoptotic protein AVEN in a preliminary small-scale yeast survival screen using the HC4 strain [38].

In parallel, we isolated high-quality mRNA (polyA^+^ RNA) from primary human tumor samples of various origins (a lung metastasis from a primary melanoma, biopsies from 6 different glioblastomas and treatment-resistant leukemic blasts isolated from 6 patients). The polyA^+^-RNA was reverse transcribed into cDNA using the SMART approach, a method that enriches for cDNAs representing full-length transcripts [31]. Size-selected cDNA (larger than 400 bp) were cloned into the constitutively active *S. pombe* expression vector, *pART1b*, which harbors a different autotrophic marker compared with the *pRIP45* vector (*leucine* instead of *adenine*). As a shuttle vector, it is also suitable for amplification in *E. coli* bacteria.

The cDNA libraries were transformed into the DSI and HC4 yeast strains, which had been previously grown in thiamine-containing repressive medium to harvest the required cell numbers. To increase the selective pressure and reduce the number of surviving yeast colonies, we transferred the DSI and HC4 cells into liquid yeast medium without thiamine 6 hours before library transformation, leading to earlier expression of *BAK* and *CED-4*. The transformed yeast cells were plated on thiamine-free agar plates to continuously induce killer protein expression. After an additional replica plating three times on thiamine-free agar plates, all non-protected yeast cells died, while a few death-resistant yeast colonies continued to grow.

The surviving yeast colonies were collected and individually expanded to isolate the transformed library plasmids, which were then directly transformed into bacteria for plasmid amplification. DNA sequencing revealed the identity of the library inserts. Occasionally, a surviving yeast colony contained two different library plasmids, in which case either insert could have been responsible for the inhibition of cell death.

### Statistical analysis of the survival screenings

**Figure 2:**
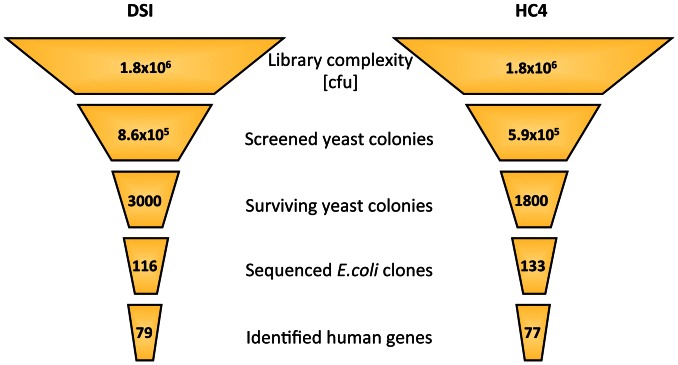
Statistics of the melanoma metastasis screen. The cDNA library prepared from a lung metastasis of a melanoma patient was transformed into the BAK (DSI) and CED-4 (HC4)-expressing yeast strains for subsequent survival screenings. Of the 8.6×10^5^ plated DSI yeast colonies, 3,000 colonies survived the killer protein expression; of these, 116 were sequenced and further analyzed. Meanwhile, from the 5.9×10^5^ plated CED-4 yeast colonies, 1,800 colonies survived the killer protein expression, and of these, 133 were sequenced and analyzed.

All three survival screens with cDNAs derived from melanoma metastasis, leukemia and glioblastoma yielded similar statistical results. Here, data from the melanoma screen are shown as a representative example (see **Fig. 2** for overview).

The overall complexity of the library was 1.8×10^6^ cfu (bacterial colony number after initial transformation of the ligated cDNA library prior to primary amplification). Transformation of the cDNA library into yeast resulted in 8.6×10^5^ DSI and 5.9×10^5^ HC4 yeast colonies, which were then screened for survival following the induction of killer protein expression. Ultimately, 3,000 (DSI) and 1,800 (HC4) surviving and growing colonies were observed on thiamine-free agar plates. We selected 116 DSI and 133 HC4 colonies for expansion and sequencing of the library inserts and identified 79 (DSI) and 77 (HC4) human genes, respectively. Fewer genes were identified than yeast colonies used for library plasmid extraction because a number of genes was identified several times in the same screen (partly with non-identical cDNA sequences; see **Table 1** and **Table S1**), including *MALAT1* from the leukemia screen (10 cDNA clones), and *B2M* from three screens (identified 6 times in glioblastoma, 6 times in melanoma with the HC4 strain, and 9 times in melanoma with the DSI strain). The latter represents one of several examples in which a gene was isolated from the same tumor-derived cDNA library in both yeast strains expressing the killer proteins CED-4 and BAK. Other examples include *HIGD1A* and *PAICS* (both found in the melanoma library). The group of genes isolated in more than one survival screen (**Table S1**) included *B2M* (found in the glioblastoma and melanoma libraries), *MALAT1* (identified in the melanoma and leukemia libraries, in both cases exclusively with the CED4-expressing HC4 yeast strain) and *SPCS2* (found in the melanoma and leukemia libraries). In addition, a few inserts represented yeast DNA instead of human (tumor-derived) cDNA. Moreover, for a limited number of sequences, BLAST searches resulted in “no significant gene alignment”.

**Table 1:** Numeric overview of the survival screen.

Gene characteristics	Number of genes	% of total
total	204	100.0
anti-apoptotic	58	28.4
upregulated in cancer	72	35.3
anti-apoptotic + upregulated in cancer	33	16.2

Gene overlap	Number of genes	% of total
multiple clone hits	41	20.1
multiple screen hits	14	6.9

As listed in the upper portion of the table, the identified genes were grouped into the following categories: total  =  number of all identified genes from melanoma metastasis, leukemia and glioblastoma screens; anti-apoptotic  =  number of isolated genes for which anti-apoptotic properties have been published; upregulated in cancer  =  number of genes that are upregulated in at least one tumor entity; anti-apoptotic + upregulated in cancer  =  number of anti-apoptotic genes upregulated in at least one tumor entity. The bottom portion of the table displays the number of genes, for which more than one clone was identified (multiple clone hits) and the number of genes for which clones were identified from at least two different cDNA libraries.

When we included the results from the leukemia and the glioblastoma screens, the results totaled 240 yeast cell death-repressing human genes, some of which were isolated in more than one of the survival screens (i.e., in different tumor entities). Without such overlap, we obtained 204 non-identical human genes found in at least one of the three survival screens that were capable of suppressing yeast cell death (the complete list of identified genes is shown in **Table S1**). As a number of genes were independently isolated several times, either in the same screen or in independent screenings with different libraries or yeast strains (**Table 1**), the results demonstrated the functional stringency of the survival screen.

Following extensive PubMed literature searches, we allocated the 204 identified genes into the following groups: 58 genes have already been described with anti-apoptotic properties, 72 genes are known to be upregulated in at least one tumor entity, and 33 genes have already been published as both anti-apoptotic and upregulated in cancer. Meanwhile, the remaining 86 genes have not been described to play a role in apoptosis and/or cancer until now. The high number of isolated genes already known to be involved in the regulation of apoptosis and cancer serves as a convincing internal control for the quality of the screen, which was designed to identify anti-apoptotic oncogenes expressed in primary tumor material.

Examples for all four groups of identified genes are shown in **Table 2**. The class of verified anti-apoptotic molecules overexpressed in cancer includes prominent tumor-associated proteins such as β-Catenin (CTNNB1) (for review see [40], [41]), Osteopontin (OPN/SPP1) (for review see [42]), BMI1 (for review see [43]), Peroxiredoxin 3 (PRDX3; [44]) and DAD1 [45]. Their anti-apoptotic and tumor-promoting influence upon (over-) activation is well established.

**Table 2:** Classification of genes isolated by yeast survival screens based on their involvement in apoptosis regulation and tumorigenesis.

Apoptosis/Cancer			
HMGB1	Osteopontin	ß-Catenin	BMI1
SGK1	CCL2	PRDX3	EIF4B
UBE2V1	NUP107	TC1	DAD1

Apoptosis								
CALM2	STAM2		ERO1L		PPP1CC			
Cancer								
PAICS		NAP1L1		STEAP1		*MALAT1*		

No link to Apoptosis/Cancer			
MAST205	NUDT5	SSR1	VPS29
FAM36A	ZCCHC10	C8orf40	PRRC1

From the 204 genes identified in the functional yeast survival screens of melanoma metastasis-, leukemia- and glioblastoma-derived cDNA libraries, a representative selection of 28 was chosen and grouped into four categories as follows: (1) “Apoptosis/Cancer” includes genes that have been described to possess anti-apoptotic properties and are implicated in tumorigenesis (minimal requirement: upregulated in tumors). (2) “Apoptosis” includes genes with published anti-apoptotic properties (but until now were not recognized to be upregulated in cancer or involved in tumorigenesis). (3) “Cancer” encompasses genes that are expressed at higher levels in tumors than in healthy samples (but have not been shown to play a role in apoptosis regulation). (4) “No link to Apoptosis/Cancer” includes genes without published links to apoptosis or cancer.

In the group of genes involved in the regulation of apoptosis but without published links to tumorigenesis, established inhibitors of apoptosis such as *PPP1CC* [46] were identified, again confirming that the yeast-based survival screen allows for the identification of tumor proteins that efficiently suppress apoptosis in human cells.

The third class of isolated yeast cell death suppressors includes molecules such as PAICS [47], STEAP1 [48]–[50] and *MALAT1* [51]–[53], for which overexpression and/or functional relevance in tumor biology has been published.

The remaining proteins have not yet been linked to tumorigenesis and/or apoptosis; although, some aspects of their physiological function are either known (e.g., MAST2 [54]–[56] and NUDT5 [57], [58]) or the molecules remain completely undescribed (e.g., FAM36A and PRRC1).

### The expression data for the selected candidate genes reveals strong and frequent overexpression in various human tumors

Literature searches have revealed that our yeast survival screen of tumor-derived cDNA libraries indeed selects for anti-apoptotic cDNAs with functional links to tumorigenesis. To assess whether some of the death-suppressing genes isolated in the survival screen are overexpressed in tumor entities, we performed a variety of tumor expression studies analyzing the three selected candidate genes. These genes were all overexpressed in certain tumor entities according to the *Oncomine* database (www.oncomine.org; the database offers gene expression analysis results based on several thousand cancer transcriptome profiles), and they displayed interesting characteristics regarding tumorigenesis and potential cancer therapy. With PAICS, we selected one enzyme of a pathway already successfully targeted by methotrexate during tumor treatment. *MALAT1* is a prominent member of the heavily investigated family of long non-coding RNAs, which is overexpressed in a variety of tumors. Finally, MAST2 is a kinase associated with microtubules and is involved in intracellular signaling: kinases represent attractive druggable targets for cancer therapy.

### PAICS

PAICS (Phosphoribosylaminoimidazole carboxylase/phosphoribosylaminoimidazole succino-carboxamide synthetase) was identified in the melanoma lung metastasis screen. The molecule is a bi-functional enzyme of the *de novo* purine biosynthesis pathway with both 5-aminoimidazole ribonucleotide carboxylase and 4-(N-succinylcarboxamide)-5-aminoimidazole ribonucleotide synthetase activities. Cancer cells rely on the PAICS-dependent metabolic pathway for AMP and GMP synthesis, while untransformed cells recover purines via the salvage pathway ([59] and references herein). Inactivation of the *de novo* pathway (e.g. by the antifolate drug methotrexate) inhibits cancer cell proliferation both *in vitro* and *in vivo* [60].

**Figure 3:**
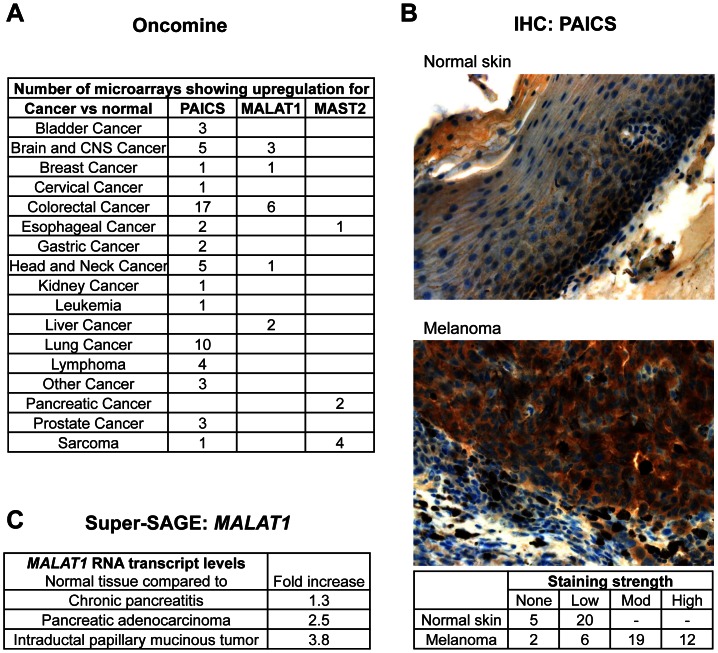
Enhanced expression of selected candidate genes in various tumor entities. **A**. mRNA expression analysis using the open access cancer microarray database *Oncomine*. The table displays the overall number of microarray analyses present in the database that show upregulation of *PAICS*, *MALAT1* or *MAST2* in the tumor type listed in the table. The *Oncomine* search was performed with the thresholds as follows: p-value < 1E-4, fold change >2, and gene rank  =  top 10%. **B**. An immunohistochemical staining of PAICS protein expression was performed with purified anti-hPAICS serum on 25 normal skin and 39 melanoma slides. Examples of a normal skin and a melanoma result are presented; the table at the bottom summarizes the results. The average expression level of PAICS protein in normal skin was low, while PAICS expression ranged between moderate (mod) and high in a majority of the melanoma biopsies. The unpaired, two-tailed Mann-Whitney test revealed significant differences between melanoma and normal skin IHC scores (p<0.0001). **C**. Super-SAGE expression analysis of normal and diseased pancreatic tissues revealed significant upregulation of *MALAT1* in chronic pancreatitis, pancreatic adenocarcinoma and intraductal papillary mucinous tumors compared with normal pancreas. The experiment was performed with RNA pools from 4 healthy pancreas samples, 3 chronic pancreatitis biopsies, 4 ductal adenocarcinomas and 5 intraductal papillary mucinous tumors.

Analysis of the *Oncomine* database demonstrated that *PAICS* mRNA is significantly overexpressed in a variety of tumor entities, including colorectal cancer, brain and CNS cancer, bladder cancer and lymphoma (**Fig. 3A**). The highest level of overexpression was observed in a squamous cell lung carcinoma (SCLC) study, with a 19-fold increase in *PAICS* mRNA expression compared with normal tissue. We then developed a peptide-derived anti-human PAICS antiserum (see **Fig. S1** for specificity control by Western blot analysis) for immunohistochemical (IHC) analysis of several tumor entities represented on a multi-tumor array. IHC analysis revealed significant PAICS protein overexpression in melanoma samples compared with normal skin biopsies; this result was confirmed when the IHC analysis was repeated with a cohort of 39 melanomas in which the PAICS expression levels were compared with 25 normal skin samples (**Fig. 3B**).

### MALAT1

*MALAT1* (metastasis associated lung adenocarcinoma transcript 1) is a long non-coding RNA of 8.7 kb that functions in mRNA splicing [61] and has been reported to be strongly overexpressed in various human solid carcinomas, including lung, breast, pancreas, colon, prostate, endometrial stromal sarcoma and HCC (for references see [53]). For stage I non-small cell lung cancers (NSCLC), high *MALAT1* expression levels have been described to correlate with metastasis [52], [62].

We isolated several *MALAT1* cDNA clones from the melanoma- and leukemia-derived tumor libraries. Our *Oncomine* search confirmed *MALAT-1* overexpression in various tumor entities (**Fig. 3A**), with the highest levels of *MALAT-1* overexpression observed in rectal mucinous adenocarcinoma (6-fold overexpression compared with normal tissue). RNA expression data obtained by Super-SAGE (serial analysis of gene expression) revealed 1.3- to 3.8-fold *MALAT-1* overexpression in chronic pancreatitis, pancreatic adenocarcinomas and intraductal papillary mucinous tumors compared with normal pancreas tissue (**Fig. 3C**).

### MAST2

Microtubule associated serine/threonine kinase 2 (MAST2/MAST205) is a 205-kDa serine/threonine kinase that is associated with microtubules [63] and plays an important role in the regulation of LPS-induced cytokine response of macrophages [56]. MAST2 has also been reported to form a complex with TRAF6, thereby resulting in the inhibition of NF-κB activation ([55] and references herein). MAST2 represents the founding member of the MAST protein family consisting of MAST1-4 [64]. All family members are characterized by the presence of the serine/threonine kinase and PDZ domains. Somatic alterations of *MAST* genes have not previously been described to play a role in cancer development. Several expression studies submitted to *Oncomine* have demonstrated *MAST2* mRNA overexpression in esophageal cancer, pancreatic cancer and sarcomas (**Fig. 3A**).

Collectively, our tumor expression analysis of *PAICS*, *MALAT-1* and *MAST2* confirmed the overexpression of all three genes in various tumor entities, thereby supporting the tumor relevance of candidate genes isolated by functional yeast survival screenings of tumor-derived cDNA libraries.

### *In vitro* downregulation of selected candidate genes in several tumor cell lines results in increased apoptosis and decreased proliferation

**Figure 4:**
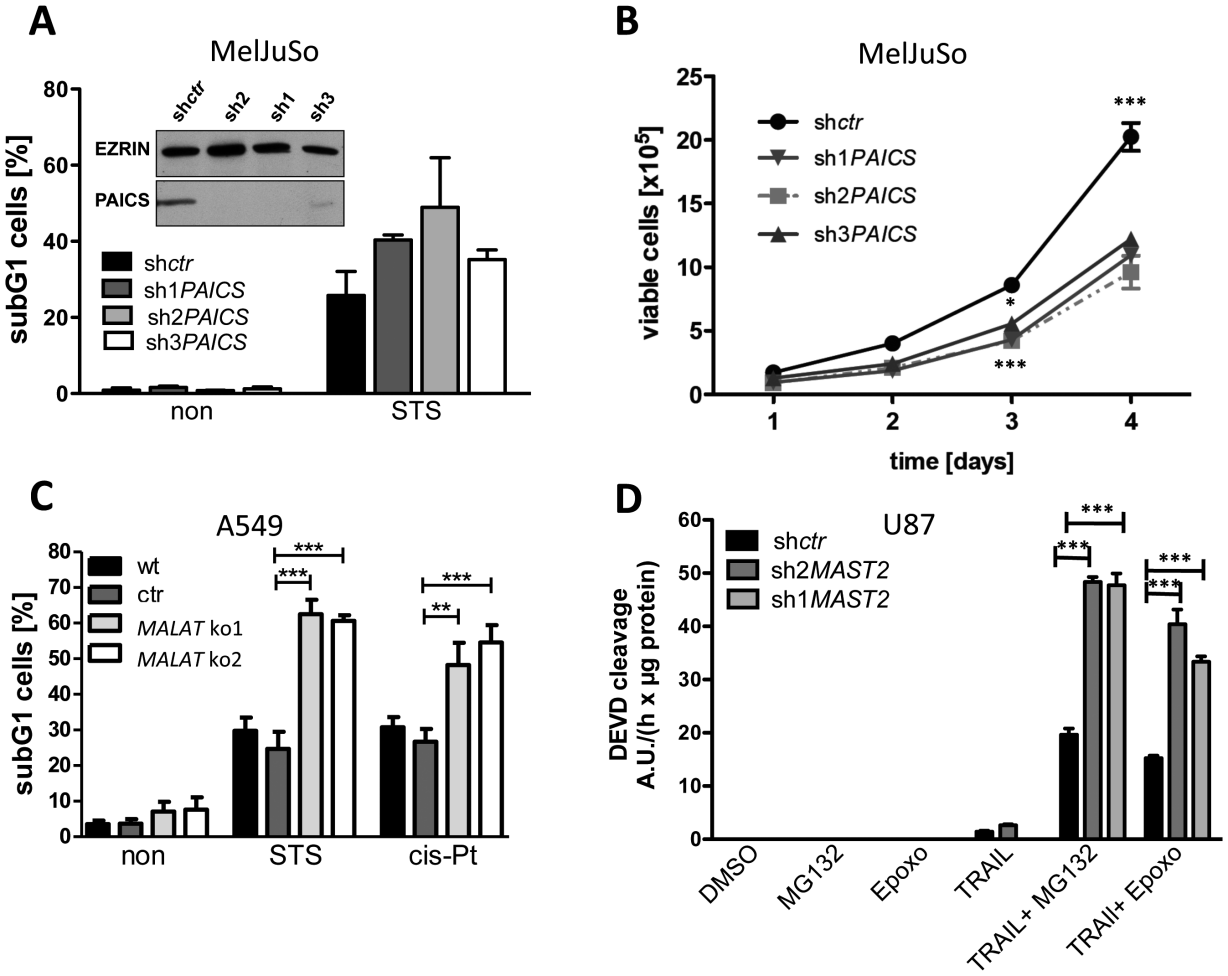
Increased apoptosis sensitivity and decreased proliferation in *PAICS*, *MALAT1* and *MAST2* knockdown cells. **A**. MelJuSo melanoma cells with stable *pGIPZ* shRNA-mediated *PAICS* knockdown (sh1-3*PAICS*) and control shRNA (sh*ctr*)-transduced cells were incubated for 24 hours with 0.2 µM staurosporine. Apoptosis was quantified by FACS using the Nicoletti protocol [36], and data are presented as the mean ± SEM, n = 4. Efficient knockdown of *PAICS* was confirmed in Western blot analysis using an anti-PAICS antiserum (inlet). **B**. Cell expansion kinetics of MelJuSo cells upon *pGIPZ* shRNA-mediated *PAICS* knockdown (sh1-3*PAICS*). Viable cells were quantified using a *CASY* cell counter, and cell numbers were compared with cells transduced with a non-targeting control shRNA (sh*ctr*). Data represent the mean values ± SEM, n = 3. One-way-ANOVA testing with Bonferroni multi-comparison correction was performed. The significance is indicated by stars for the comparison of sh*ctr* versus sh1-3*PAICS* (*: p-value <0.05; ***: p-value <0.001). The *PAICS* knockdown efficiencies were analyzed via immunoblotting (see inlet **Fig. 4A**). **C**. Apoptosis assays using the Nicoletti FACS protocol were performed with parental A549 cells (wt), control GFP cells (ctr) and two zinc finger nuclease technology (ZFN)-mediated *MALAT1* knockout cell clones (ko1 and ko2) [30]. Results are shown for both untreated cells and cells incubated for 16 hours with either 1 µM staurosporine or 400 µM cisplatin. Data are presented as the mean ± SEM (n = 6), One-way-ANOVA testing with Bonferroni multi-comparison correction was applied, and statistical significance is indicated as **: p<0.01, ***: p<0.001. Confirmation of the *MALAT1* knockout is presented in **Fig. S6A**. **D**. Two stable *pGIPZ*-mediated *MAST2* knockdown cell lines were established from parental U87 cells (for evaluation of the knockdown efficiencies, see **Fig. S6B**), and following incubation of the cells with DMSO (solvent control), recombinant TRAIL (250 ng/µl), MG132 (2,5 µM), epoxomicin (50 nM), TRAIL plus MG132, and TRAIL plus epoxomicin for 16 h, apoptosis was quantified in a CASPASE-3 activity assay. Control shRNA-transduced U87 cells served as a control. Data are presented as the mean ± SEM (n = 4), One-way-analysis of variance (ANOVA) testing with Bonferroni multi-comparison correction was applied, and statistical significance is indicated as ***: p<0.001.

Because we isolated PAICS from the melanoma metastasis cDNA library and found PAICS protein levels to be upregulated in melanomas, we targeted PAICS expression in the human melanoma cell line MelJuSo by lentiviral transduction with three different *PAICS* shRNA sequences cloned into the *pGIPZ* vector. MelJuSo and other established melanoma cell lines express prominent amounts of PAICS protein exceeding the PAICS levels observed in the spontaneously immortalized human keratinocyte cell line HaCaT and in human foreskin fibroblast cells (see **Fig. S2A**). Efficient PAICS downregulation in MelJuSo cells by the shRNAs was confirmed by Western blot analysis after puromycin selection (see inlet in **Fig. 4A**). As shown in **Fig. 4A**, all MelJuSo *PAICS* knockdown cell lines showed increased levels of staurosporine-induced apoptosis compared with control shRNA-transduced cells. MelJuSo cells transduced with sh2*PAICS* shRNA were then analyzed in further cell death assays following apoptosis induction by various apoptotic stimuli. Treatment of *PAICS* knockdown in MelJuSo cells with cisplatin, mitomycin C, staurosporine and etoposide led to increased apoptosis in comparison to control shRNA-transduced cells (**Fig. S3A**). For cisplatin and staurosporine, this difference was statistically significant.

We then used the *PAICS* knockdown MelJuSo cell lines for a cell counting experiment. **Fig. 4B** shows significantly lower cell expansion rates for all three *PAICS* shRNA MelJuSo cell lines compared to the control shRNA-transduced cells. As no significant difference in the apoptosis rate of untreated *PAICS* and control shRNA-transduced cells was observed (**Fig. 4A**), this difference in cell expansion may be attributed to decreased proliferation rather than increased apoptosis.

In the absence of *puromycin* selection, the knockdown of *PAICS* vanished with increasing numbers of passages, indicating a strong selective pressure against the loss of PAICS in the cells. Therefore, we introduced a doxycycline-inducible *PAICS* shRNA system (using the sh2*PAICS* shRNA sequence) into MelJuSo cells, which allowed for a complete knockdown of *PAICS* mRNA following 3 days of doxycycline treatment. Six days after removal of doxycycline from the medium, the cells expressed normal PAICS levels (**Fig. S4**). MelJuSo cells expressing the inducible *PAICS* knockdown vector system were again used for a cell counting experiment. The increase in viable cells was identical in non-induced *PAICS*- and control shRNA-transduced cells in the absence of doxycycline. However, upon the knockdown of *PAICS* in the presence of doxycycline, the cells expanded significantly slower and almost stopped proliferating (see **Fig. S3B**).

To further investigate the tumorigenicity of the *PAICS*-deficient MelJuSo cells and analyze the influence of PAICS on anchorage-independent growth, we seeded MelJuSo cells with wildtype PAICS level or cells with induced *PAICS* knockdown in semi-solid methyl cellulose and subsequently quantified colony growth after 6 days. We performed this assays with both untreated healthy cells and cells treated with staurosporine. In both cases, the colony numbers were significantly reduced in the absence of PAICS, thereby confirming that PAICS plays an important role in the tumorigenicity of melanoma cells (see **Fig. S5A**).

*MALAT1* expression was originally associated with metastasis in NSCLC patients and described as an adverse prognostic parameter for patient survival during stage I NSCLC [52], [62]. We selected the human lung carcinoma cell line, A549, to investigate the *in vitro* consequences of *MALAT1* deficiency for apoptosis sensitivity and proliferation potential. A549 cells express elevated *MALAT1* RNA levels, even higher than the mRNA levels produced by the house keeping gene *GAPDH* [52], [62]. Knockdown via RNAi is insufficient for such levels of nuclear ncRNA. In previous work with several *MALAT1* siRNA sequences, we have achieved a knockdown efficiency of 13% residual *MALAT1* RNA levels [30]. However, because of the high *MALAT1* expression levels in A549 cells, the substantial residual expression of *MALAT1* could mask its loss-of-function phenotype and prevent the functional characterization of *MALAT1*. To generate *MALAT1*-deficient cells for functional analyses, we used a zinc-finger nuclease (ZFN) and integrated RNA-destabilizing elements (RDE) into the human *MALAT1* gene locus [30]. Biallelic RDE integration resulted in a 1000-fold reduction in RNA expression levels, which represents a far greater reduction compared with that achieved by shRNA-mediated knockdown of the abundant *MALAT1* molecules in A549 cells (**Fig. S6A**). Interestingly, *MALAT1* deficiency led to a significant increase in apoptosis sensitivity in A549 cells treated with staurosporine and cisplatin (**Fig. 4C**), which correlates with an apoptosis-inhibiting function of *MALAT1*. However, absence of *MALAT1* did not influence the expansion of cells, thereby suggesting that proliferation was not affected by removal of *MALAT1* (data not shown).

To perform an additional cell culture-based assessment of the tumorigenicity of cells in either the presence or absence of *MALAT1* RNA, we seeded GFP-expressing control A549 cells and the two ZFN-mediated A549 *MALAT1* knockout cell clones at a low density and subsequently counted the colonies after 8 days using crystal violet staining. As shown in **Fig. S5B**, the knockout of *MALAT1* resulted in a significant decrease in clonogenicity and colony size, thereby suggesting an important function of *MALAT1* in sustaining the transformed phenotype of the tumor cells.

*MAST2* was isolated from the glioblastoma cDNA library. The protein is significantly expressed in the glioblastoma cell line U87, whereas MAST2 expression is absent in human cortex tissue, which consists of many astrocytes, probably constituting the cells-of-origin for glioblastoma (see **Fig. S2B**). To investigate its oncogenic potential *in vitro*, we suppressed *MAST2* expression in U87 cells via lentiviral shRNA transduction and subsequently selected for two stable knockdown cell lines (*MAST2* mRNA expression levels are presented in **Fig. S6B**). Following co-treatment with TRAIL and two different proteasome inhibitors (a promising combination for future glioblastoma therapy [65]), the apoptotic profile of *MAST2* shRNA U87 cells was assessed and we observed a significant increase in apoptosis sensitivity in the absence of *MAST2* (**Fig. 4D**), thereby confirming the anti-apoptotic potential of this kinase. We also noticed a decrease in cell proliferation upon knockdown of *MAST2* as measured by quantification of EdU incorporation during DNA synthesis (**Fig. S7**; note that the U87 *MAST2* knockdown cell line with the lower amount of remaining *MAST2* mRNA proliferated even less than the second *MAST2* knockdown cell line). Both the anti-apoptotic and pro-proliferative molecular functions observed suggest that MAST2 behaves as an oncoprotein in glioblastoma.

### Targeting PAICS and MAST2 in an *in vivo* xenograft mouse model leads to diminished tumor growth

To obtain further experimental *in vivo* evidence that the candidate genes isolated in the functional yeast survival screen play an important oncogenic role in tumor progression, we utilized both the U87 *MAST2* and inducible MelJuSo *PAICS* knockdown tumor cell lines (used previously in cell culture assays) in a xenograft tumor mouse model.

**Figure 5:**
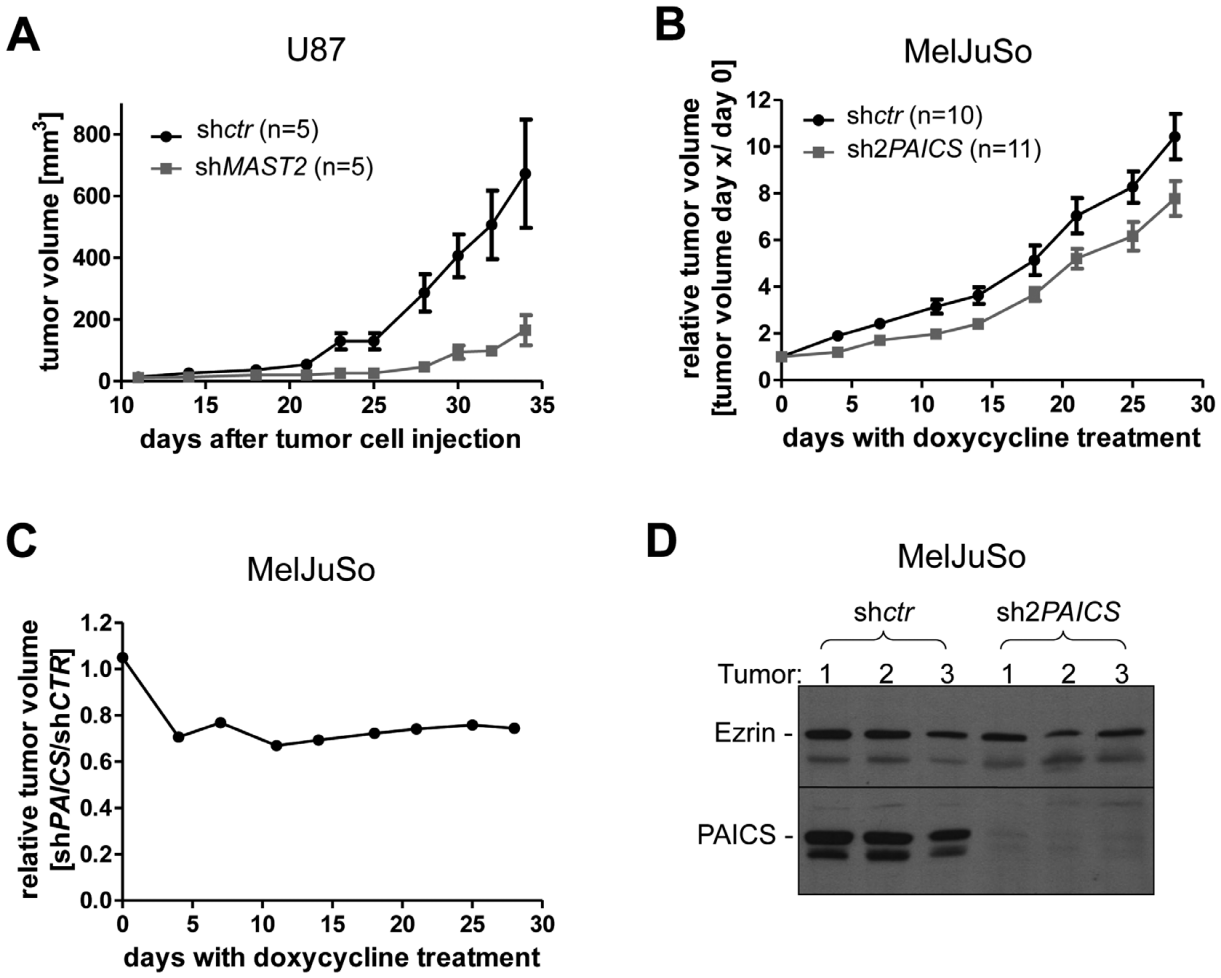
Xenograft tumor growth of *MAST2* and *PAICS* knockdown in U87 and MelJuSo cells, respectively. **A**. The subcutaneous xenograft tumor growth of stable sh2*MAST2* knockdown U87 cells was quantified with a manual caliper 2-3 times per week and compared with non-targeting control shRNA (sh*ctr*)-transduced cells. Data are presented as the mean ± SEM. 5 mice/group were injected into the right flank. The unpaired, two-tailed t-test was performed individually for each day after injection of the tumor cells and revealed the following p-values: day 23, 0.0063; day 25, 0.0063; day 28, 0.0169; day 30, 0.0127; day 32, 0.0219; day 34, 0.0496. **B**. *pTRIPZ* sh2*PAICS*-transduced MelJuSo were injected subcutaneously into the right flank of NOD-SCID mice. When the xenografted tumors reached a size of 50 mm^3^, mice received drinking water containing 2 mg/ml doxycycline and 10 g/l sucrose *ad libitum* until the end of the experiment to induce the *PAICS* shRNA knockdown. The graph displays the relative tumor growth (tumor volumes from day x/day 0 of doxycycline treatment). Injected mice per group: sh*ctr*  = 10, sh2*PAICS*  = 11. Data are presented with the mean values and error bars indicate SEM. The unpaired, two-tailed t-test was performed individually for each day after the start of doxycycline treatment and revealed significant differences for day 4 to day 28 with doxycycline treatment (day 4, 0.0028; day 7, 0.0163; day 11, 0.0024; day 14, 0.0082; day 18, 0.0414; day 21, 0.0429; day 25, 0.0311; day 28, 0.0414). In **C**, the relative tumor size is shown (calculated as tumor volume of sh2*PAICS* MelJuSo cells divided by tumor volume of sh*ctr* cells). **D**. Three xenograft tumors per shRNA used (sh*ctr*, sh2*PAICS*) were isolated, and PAICS protein levels were analyzed via Western blot. In contrast to the MelJuSo cells grown in cell culture (see **Fig. 4B**), an additional 40-kDa band appeared in the anti-PAICS Western Blot when the lysates were prepared from MelJuSo tumors grown in mice. As with the original 37-kDa PAICS band, this larger signal disappeared in the *PAICS* knockdown cells, thereby arguing that it represents a posttranslationally modified PAICS isoform.

When we injected U87 cells stably transduced with either sh2*MAST2* or control shRNA subcutaneously into the right flank of immunodeficient NOD/SCID mice, we observed a significant delay in tumor growth in the sh2*MAST2* animals (see **Fig. 5A**). This observation demonstrates that MAST2 plays an important role in glioblastoma growth by suppressing apoptosis and increasing cellular proliferation.

We also analyzed the consequences of PAICS deficiency in MelJuSo melanoma cells using a subcutaneous xenograft tumor model. Because the constitutive *PAICS* knockdown was unstable in MelJuSo cells (see above), we used cells with a doxycycline-inducible *PAICS* knockdown for the xenograft experiment. Tumors were grown without *PAICS*-specific shRNA expression until they reached a volume of 50 mm^3^. The mice then received drinking water supplemented with doxycycline to induce *PAICS* knockdown. **Fig. 5B** shows the relative tumor growth for *PAICS* and control knockdown cells in relation to the tumor volume at the beginning of doxycycline treatment. The *PAICS* knockdown tumors grew significantly slower compared with the control cells with normal PAICS expression. **Fig. 5C** displays the relative tumor size calculated as the tumor volume of the *PAICS* knockdown tumors divided by the tumor volume of the control shRNA-transduced cells. The data suggest that the differences in tumor growth shown in **Fig. 5B** are primarily caused by an initial arrest of tumor growth directly following the induction of the *PAICS* knockdown. However, after day 5 of doxycycline treatment, the *PAICS* and control knockdown tumors grew with comparable kinetics (horizontal progression of the curve in **Fig. 5C**). The *PAICS* knockdown tumors failed to re-express the PAICS protein as shown in **Fig. 5D**. Interestingly, the tumor cells quickly adapted to growth in the absence of PAICS, possibly by switching to the salvage pathway for purine biosynthesis.

## Discussion

In recent years, the concept in cancer therapy to specifically target tumor cells while sparing the untransformed normal cells has gained much attraction. This is not only due to reduced side effects for the patient and higher success rates, but also because immune effector cells remain unaffected by cytotoxicity and can help to eradicate the tumor [66]. A better understanding of all deregulated molecular players that induce oncogenic signaling in tumors would enhance future therapeutic options by denoting new targets. Moreover, the successful analysis of their function will allow for the application of specified therapeutics such as personalized cancer treatment [67].

Tumor cells survive by withstanding a plethora of different apoptosis stimuli. The acquirement of apoptosis resistance by loss-of-function (pro-apoptotic tumor suppressors) or gain-of-function (anti-apoptotic oncoproteins) mutations is an important hallmark of tumorigenesis [7]. Therefore, the interference with anti-apoptotic signaling in tumor cells is considered a promising cancer therapy strategy [68]. The functional yeast survival screen described in this manuscript exploits the genetically well-defined yeast system to screen cDNA libraries in a high throughput format for novel anti-apoptotic proteins. In principle, the screen can be adapted to identify apoptosis inhibitors of a variety of biological systems. By selecting the appropriate killer protein to induce yeast cell death, as well as generating and screening cDNA libraries from the relevant tissue or cellular source, anti-apoptotic proteins regulating various biological processes, such as embryonic development or tissue homeostasis in the adult organism, can be isolated. Another interesting application is the identification of (deregulated) apoptosis inhibitors from diseased tissues (cancer, infection or auto-immunity). Here, we used cDNA libraries prepared from various tumor entities (lung metastasis of a primary melanoma, glioblastoma and leukemic blasts) to identify novel anti-apoptotic oncoproteins that are involved in tumorigenesis and/or therapy resistance. We also performed proof-of-principle experiments to evaluate a few of the identified apoptosis regulators as molecular cancer therapy targets.

Of the 204 genes identified in the three screens, 28% were already known to inhibit apoptosis according to the literature, 35% were described as upregulated in expression databases in at least one tumor entity, and 16% of the genes were described both as anti-apoptotic in function and overexpressed in tumors (**Table 1**). These genes represent the class of molecules we aimed to identify and they served as a positive control, validating the screening system. Furthermore, 42% of the isolated genes had not been previously described to play a role in apoptosis or tumorigenesis; these genes represented the pool of potential novel anti-apoptotic oncoproteins. These results demonstrate the power of the functional survival screen to identify tumor-relevant apoptosis inhibitors.

We decided to analyze the anti-apoptotic potential and the oncogenic properties of three of the identified candidate genes, *PAICS*, *MALAT1* and *MAST2*. Whereas PAICS [47] and *MALAT1* [51] have already been shown to be involved in tumorigenesis, there are no publications describing the involvement of MAST2 in apoptosis regulation or cancer.

Our *Oncomine* database searches revealed an overexpression of *PAICS*, *MALAT1* and *MAST2* mRNA in various tumor entities. According to the literature, *MALAT1* is strongly overexpressed in a variety of tumor types, including hepatoblastomas [69], hepatocellular carcinomas [53], [70], breast cancer [71], and endometrial stromal sarcomas [72]. It has also been described as a metastasis-associated factor in NSCLC and colorectal cancer [52], [73]. *Oncomine* database entries revealed that *MALAT1* overexpression has been observed in brain and CNS cancer as well as head and neck cancer. We isolated *MALAT1* from our screens of melanoma metastasis- and leukemic blasts-derived cDNA libraries, while Super-SAGE expression analysis of the long non-coding *MALAT1* RNA expression profile detected a 4-fold increase in pancreas carcinoma compared with normal tissue.

Upregulation of PAICS has been reported for lung squamous cell carcinoma [47], and our *Oncomine* database research yielded several expression studies in various tumor entities (e.g., brain and CNS cancer, bladder cancer, colorectal cancer, head and neck cancer and lung cancer) in which *PAICS* mRNA was upregulated compared with corresponding normal tissue. We isolated *PAICS* from a screen of the melanoma metastasis-derived cDNA library in both yeast strains expressing CED-4 and BAK. We subsequently confirmed that PAICS protein is overexpressed in melanomas when compared with normal skin using an anti-PAICS antiserum.

Although MAST2 has not been previously associated with apoptosis regulation and tumorigenesis, according to the *Oncomine* database, *MAST2* mRNA is overexpressed in esophageal cancer, pancreatic cancer and sarcoma samples.

Our *in vitro* cell culture experiments confirmed the anti-apoptotic potential of all three candidate genes. Stable knockdown of *PAICS* increased the sensitivity of melanoma cells to cisplatin and staurosporine treatment. Moreover, using stable and inducible *PAICS* knockdown systems in MelJuSo cells, we observed a marked decrease in cell expansion following PAICS depletion. The mechanism by which an enzyme of the *de novo* purine biosynthesis pathway influences apoptosis sensitivity at the molecular level remains elusive. The expression levels of several key apoptosis regulators (CASPASE-3, CASPASE-9, BAX, BAK, BCL-2, BCL-X_L_ and AIF) did not change upon the induced knockdown of *PAICS* (**Figure S8**). The zinc finger nuclease-mediated knockout of *MALAT1* led to a marked increase in apoptosis sensitivity in A549 lung cancer cells following incubation with staurosporine and cisplatin. Stable knockdown of *MAST2* resulted in significantly enhanced cell death in U87 glioblastoma cells upon co-treatment with TRAIL and the proteasome inhibitors MG132 and epoxomicin. These data confirm the potential of the survival screen to isolate cDNAs that inhibit cell death in tumor cells.

The xenograft tumor mouse model data supports the notion that MAST2 protein is required for tumor progression, possibly because of its anti-apoptotic potential. Stable knockdown of *MAST2* in U87 cells strongly diminished tumor growth in immunocompromised mice (**Fig. 5A**). Interestingly, during preparation of this manuscript, recurrent gene rearrangements involving *MAST2* and *MAST1* were identified in breast cancer cell lines and tissues [74]. The overexpression of these *MAST2* and *MAST1* gene fusions displayed a proliferative effect; however, the function and (tumor) biology of the wildtype MAST proteins were not further investigated. Our data demonstrate an increase in apoptosis sensitivity and diminished tumor growth upon *MAST2* knockdown in glioblastoma cells, thereby suggesting an oncogenic function for this poorly analyzed kinase.

The induced knockdown of *PAICS* in MelJuSo melanoma cells also decreased tumor xenograft growth; however, the difference compared with tumor growth of the parental cell line with endogenous PAICS expression was rather small (**Fig. 5B**). Apparently, the tumor cells stopped proliferating for the first couple of days following PAICS depletion before they resumed expansion (**Fig. 5C**). Analysis of the outgrown tumors revealed that the tumor cells maintained a stable *PAICS* knockdown. It is quite likely that the MelJuSo cells switch back to the salvage pathway for purine biosynthesis following the blockage of the *de novo* synthesis pathway. Based on these results, the bi-functional PAICS enzyme might only represent a reasonable target for molecular therapies in certain tumors, possibly those with an inactivated/mutated salvage pathway for purine biosynthesis.

We also performed xenograft tumor experiments with *MALAT1* knockdown A549 lung cancer cells. However, we did not observe significant differences in tumor growth without further treatment, thereby arguing against a significant involvement of *MALAT1* in A549 primary tumor growth. Nevertheless, we confirmed that *MALAT1* plays an important role in tumor metastasis using an A549 metastasis mouse model [75].

In summary, our data confirm the power of the functional yeast survival screen to isolate novel anti-apoptotic genes regulating cell death [18]–[21]. The success of the screen is based on several characteristics. As proteins regulating programmed cell death are functionally conserved among species, the well-characterized yeast genetics can be used to screen in a high throughput modus. The screen is also based on functional readout (survival) and can be customized by selecting the appropriate killer protein and cDNA library. With these characteristics, it can be adapted to answer a variety of (patho-) biological questions involving the identification of programmed cell death inhibitors.

## Supporting Information

Figure S1 **Validation of self-raised rabbit anti-PAICS antiserum.** Two peptides derived from human PAICS (aa 10–23 and aa 222–235) were simultaneously used for rabbit immunization. The antigen-purified anti-PAICS serum was employed for Western blot analysis of cell lysates from HEK 293T and RKO cells, which had been transduced with either *pSIEW*-PAICS or *pSIEW-Flag*-*PAICS* (**A**, B; non-transduced cells served as a control for endogenous PAICS) or with the empty vector control (293T *pSIEW*; **C**). The anti-PAICS antiserum recognizes both endogenous PAICS and overexpressed Flag-PAICS protein (**A** and **B**), and the latter was confirmed using anti-Flag antibody (**C**). Anti-EZRIN served as a loading control.
(TIF)

Figure S2 **Relative expression levels of PAICS and MAST2 in MelJuSo and U87 cells.** **A**. Western blot analysis of PAICS protein levels in MelJuSo and other melanoma cell lines compared to primary human foreskin fibroblasts (HFFC) and the immortalized normal keratinocyte cell line HaCaT. Following protein transfer, the membrane was stained with Ponceau S to confirm equal protein loading, prior to incubation with the self-raised anti-PAICS antiserum. **B**. Immunhistochemical analysis of MAST2 protein levels in human cortex (left panel; scale bar  = 100 µm) and U87 cells (right panel). In normal human brain samples, MAST2 expression was virtually absent from both neuronal and glial cells. Most U87 glioma cells show moderate to strong MAST2 expression.
(TIF)

Figure S3 **Increased apoptosis and decreased proliferation in MelJuSo cells upon** ***PAICS*** **knockdown.** **A**. MelJuSo melanoma cells with stable *pGIPZ* sh2RNA-mediated *PAICS* knockdown and control shRNA (sh*ctr*)-transduced cells were analyzed in apoptosis assays. Untreated cells and cells incubated for 24 hours with 400 µM cisplatin, 50 µM etoposide, 0.2 µM staurosporine or 5 µg/ml mitomycin C were analyzed by FACS using the Nicoletti protocol [36]. Data are presented as the mean ± SEM, n = 3 (paired, two-tailed t-test, **: p-value<0.01). The *PAICS* knockdown was confirmed by Western blot analysis (see inlet of **Fig. 4A**). **B**. Cell growth of MelJuSo cells upon initiation of a *pTRIPZ* sh2RNA-mediated inducible *PAICS* knockdown. Viable cells were quantified using a *CASY* cell counter. All cells were incubated with doxycycline to induce shRNA expression, and cell numbers were compared with parental non-transduced wildtype cells as well as to cells transduced with a non-targeting control shRNA (sh*ctr*). Data represent the mean values with SEM, n = 3. One-way-ANOVA testing with Bonferroni multi-comparison correction was performed. The significance is indicated by asterisks for the comparison of sh*ctr* versus sh2*PAICS* (***: p-value<0.001). PAICS protein levels were assessed via immunoblotting of cells with (Doxy +; sh*PAICS* expressed) and without (Doxy -; no sh*PAICS* expression) doxycycline treatment. Anti-Ezrin served as a loading control. *ctr*: cells transduced with control shRNA; *kd*: cells transduced with *pTRIPZ* sh2*PAICS*.
(TIF)

Figure S4 **Inducible** ***PAICS*** **knockdown in MelJuSo cells.** MelJuSo cells were transduced with the inducible shRNA vector *pTRIPZ* sh2*PAICS*. Doxycycline treatment induced the expression of both the turbo red fluorescent protein (tRFP) and *PAICS*-specific shRNA. **A** depicts images of *pTRIPZ* sh2*PAICS*-transduced MelJuSo cells after three days of doxycycline treatment (+3d) and images of the same cell culture after six days of doxycycline withdrawal (−6d). The upper panel represents transmitted-light images, while the lower panel displays red fluorescence images showing tRFP expression. **B**. A Western blot membrane incubated with anti-PAICS antiserum, which visualizes *PAICS* protein levels in cells without shRNA induction by doxycycline, and after both three and six days of incubation with 1 µg/ml doxycycline (induction of shRNA expression). Cell lysates prepared after three and six days of doxycycline withdrawal were also analyzed. Equal protein loading was confirmed by anti-EZRIN staining.
(TIF)

Figure S5 **Colony formation assays with** ***PAICS*** **knockdown and** ***MALAT1*** **knockout tumor cell lines. A**. After pre-treatment for 3 days in medium with 1 µg/ml doxycycline, 2.5×10^3^ MelJuSo cells stably transduced with *pTRIPZ* sh*2PAICS* or sh*ctr* were seeded in methyl cellulose containing 40% RPMI medium (supplemented with 30% FCS, 3% penicillin/streptomycin and 3 µg/ml doxycycline). For the induction of apoptosis, the cells were incubated with 0.2 µM staurosporine 16 hours before seeding. Colony growth was quantified after 6 days. **B**. A549 cells (7.5x10^2^ cells per well) were plated in normal DMEM/10% FCS medium in triplicate in 6-well plates, and the colonies were allowed to grow for 8 days. GFP control cells were compared with the zinc-finger nuclease-mediated *MALAT1* knockout clones, ko1 and ko2. The colonies were fixed and stained with crystal violet solution and counted in a 3×3 cm scoring grid. The experiments were performed three times, and the results are represented as mean ± SEM. *p<0.05; **p<0,01; one-way ANOVA analysis with Bonferroni multi-comparson correction.
(TIF)

Figure S6 **Quantitative PCR analysis of A549-ZFN-** ***MALAT1*** **knockout and** ***MAST2*** **shRNA knockdown U87 cells. A**. The residual *MALAT1* RNA levels expressed in A549-ZFN-*MALAT1*-knockout cells (ko1 and ko2) were compared with both control (*ctr*; cells with random *GFP* integration) and parental A549 (wt) cells by Real Time qPCR analysis. Data are presented as mean values ± SEM (n = 3) on a logarithmic scale (log10). **B**. The lentiviral *MAST2* shRNA knockdown in U87 cells with two different *MAST2* shRNA sequences was confirmed via qPCR in comparison with non-targeting control shRNA-transduced U87 cells. Relative *MAST2* mRNA expression levels are presented from a single experiment. *GAPDH* and *HPRT* mRNA expression were used for relative quantification of *MAST2* expression.
(TIF)

Figure S7 **Quantification of proliferation upon shRNA-mediated knockdown of** ***MAST2*** **.** The two U87 *MAST2* knockdown cell lines sh1*MAST2* and sh2*MAST2* were compared to control vector-transduced cells for their proliferation rate using the Click-iT® Edu Proliferation Assay kit (Alexa Fluor 488, Life Technologies). Cells were incubated with EdU for one hour, and the percentage of EdU-positive cells was quantified by FACS analysis. Data are presented as the mean values ± SEM (sh2*MAST2*: n = 3; sh1*MAST2*, sh*ctr*: n = 4).
(TIF)

Figure S8 **Expression levels of several apoptosis regulators in either the presence or absence of PAICS.** The melanoma cell line, MelJuSo, was stably transduced with the lentiviral construct, *pTRIPZ* sh2*PAICS*, and the downregulation of PAICS was induced using doxycycline. 50 µg (per lane) of protein lysate was loaded to the gel and Western Blot analyses were performed either as described in the manuscript (for CASPASE-9) or using the Odyssey system (for all other proteins). The following antibodies were used: self-raised anti-PAICS antiserum (see manuscript), anti-CASPASE-3 (Cell Signaling, #9662), anti-CASPASE-9 (Alexis, #ALX-210-838-R100), anti-BAX (Upstate, #06-499), anti-BAK (Santa Cruz, #sc832, clone G-23), anti-BCL-x_L_ (Cell Signaling, #2764, clone 54H6), anti-BCL-2 (Santa Cruz, #sc509, clone 100), anti-AIF (Chemicon, #AB16501), anti-PARP (Cell Signaling, #9542) and anti-GAPDH (Calbiochem, #CB1001). The following secondary antibodies were used with the Odyssey system: IRDye 680CW goat anti-rabbit (LI-COR, #926-32221) and IRDye 800CW goat anti-mouse (LI-COR, #926-32210).
(TIF)

Table S1 Complete list of genes identified by functional yeast survival screening of cDNA libraries derived from a melanoma metastasis, leukemia samples and glioblastomas.
(PDF)
