# Supplementary figures and images for: A Functional Yeast Survival Screen of Tumor-Derived cDNA Libraries Designed to Identify Anti-Apoptotic Mammalian Oncogenes
Source: PLoS One. 2013 May 22;8(5):e64873. doi: 10.1371/journal.pone.0064873 (PMC3661464; doi:10.1371/journal.pone.0064873)

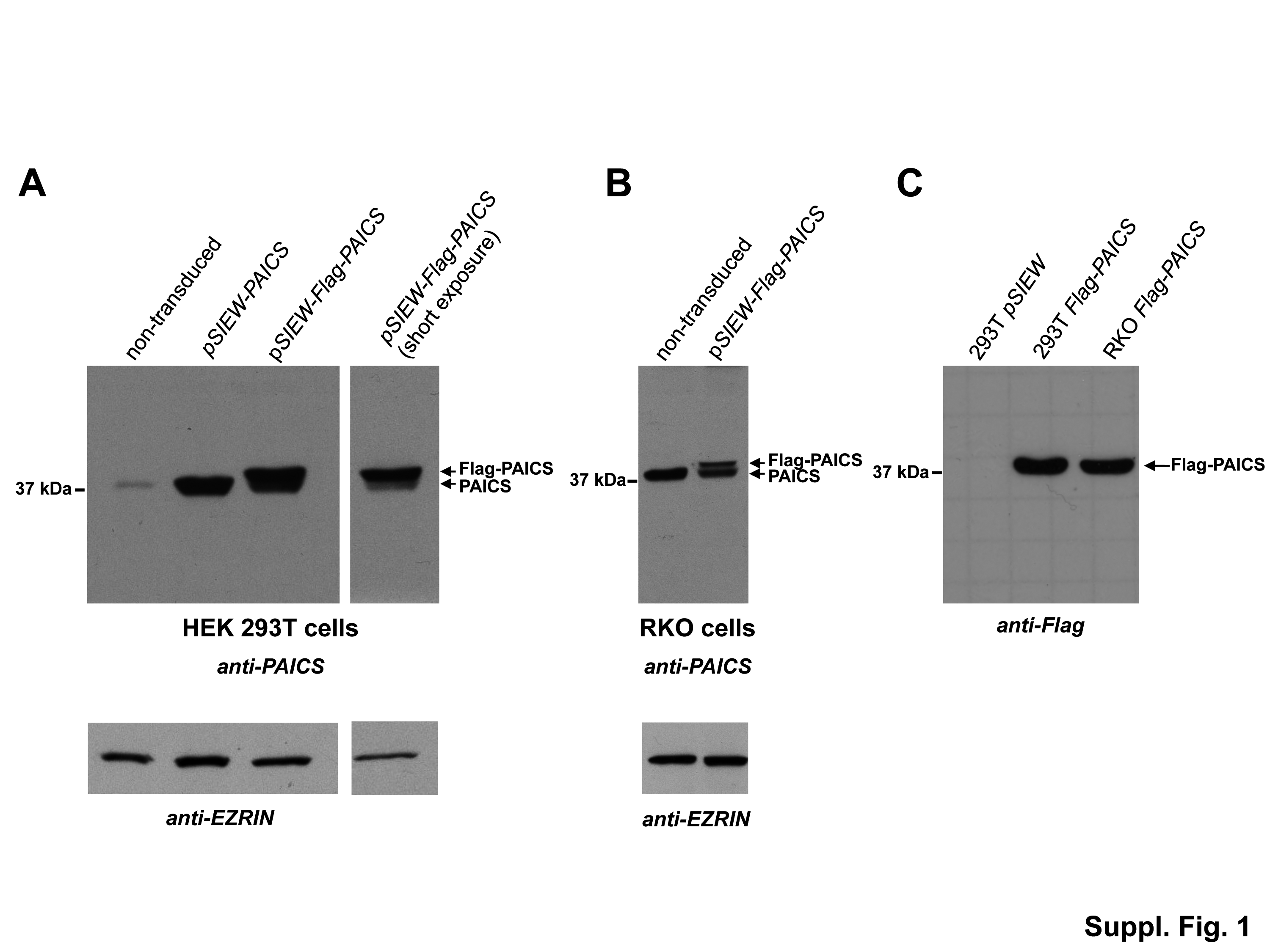

Supplement: Figure S1 — Validation of self-raised rabbit anti-PAICS antiserum. Two peptides derived from human PAICS (aa 10–23 and aa 222–235) were simultaneously used for rabbit immunization. The antigen-purified anti-PAICS serum was employed for Western blot analysis of cell lysates from HEK 293T and RKO cells, which had been transduced with either pSIEW-PAICS or pSIEW-Flag-PAICS (A, B; non-transduced cells served as a control for endogenous PAICS) or with the empty vector control (293T pSIEW; C). The anti-PAICS antiserum recognizes both endogenous PAICS and overexpressed Flag-PAICS protein (A and B), and the latter was confirmed using anti-Flag antibody (C). Anti-EZRIN served as a loading control. (TIF) [file pone.0064873.s001.tif]

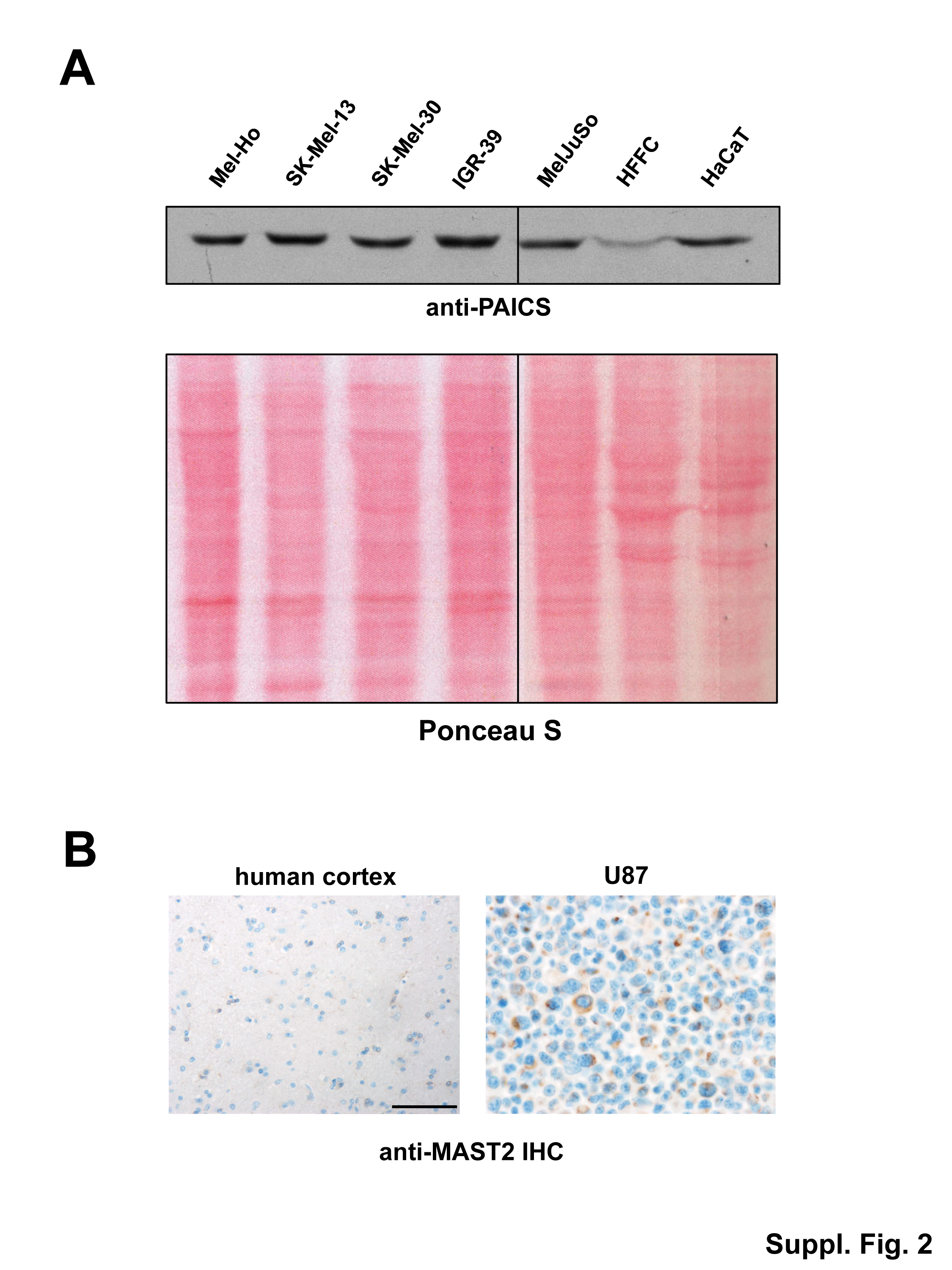

Supplement: Figure S2 — Relative expression levels of PAICS and MAST2 in MelJuSo and U87 cells. A. Western blot analysis of PAICS protein levels in MelJuSo and other melanoma cell lines compared to primary human foreskin fibroblasts (HFFC) and the immortalized normal keratinocyte cell line HaCaT. Following protein transfer, the membrane was stained with Ponceau S to confirm equal protein loading, prior to incubation with the self-raised anti-PAICS antiserum. B. Immunhistochemical analysis of MAST2 protein levels in human cortex (left panel; scale bar = 100 µm) and U87 cells (right panel). In normal human brain samples, MAST2 expression was virtually absent from both neuronal and glial cells. Most U87 glioma cells show moderate to strong MAST2 expression. (TIF) [file pone.0064873.s002.tif]

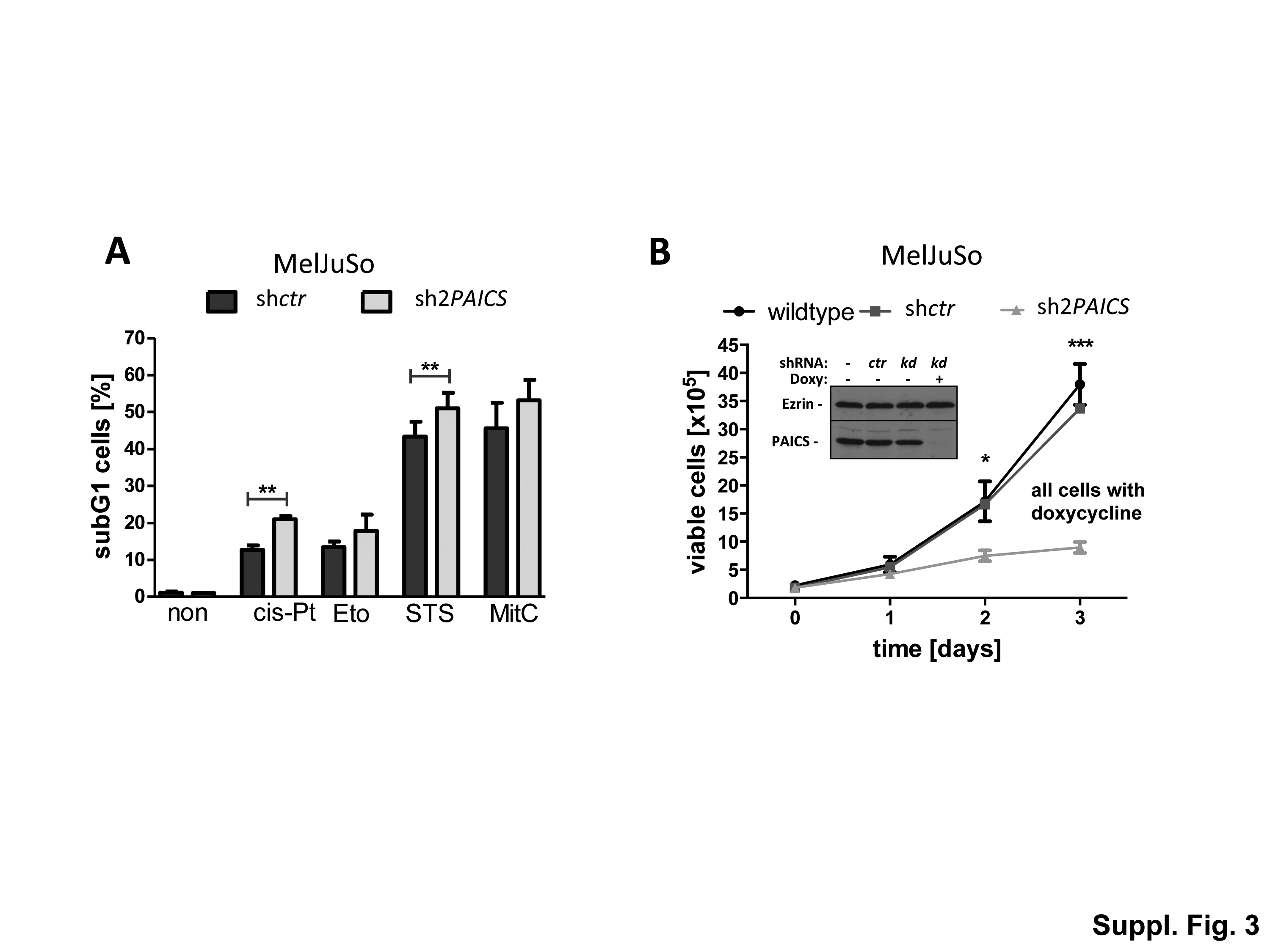

Supplement: Figure S3 — Increased apoptosis and decreased proliferation in MelJuSo cells upon PAICS knockdown. A. MelJuSo melanoma cells with stable pGIPZ sh2RNA-mediated PAICS knockdown and control shRNA (shctr)-transduced cells were analyzed in apoptosis assays. Untreated cells and cells incubated for 24 hours with 400 µM cisplatin, 50 µM etoposide, 0.2 µM staurosporine or 5 µg/ml mitomycin C were analyzed by FACS using the Nicoletti protocol [36]. Data are presented as the mean ± SEM, n = 3 (paired, two-tailed t-test, **: p-value<0.01). The PAICS knockdown was confirmed by Western blot analysis (see inlet of Fig. 4A ). B. Cell growth of MelJuSo cells upon initiation of a pTRIPZ sh2RNA-mediated inducible PAICS knockdown. Viable cells were quantified using a CASY cell counter. All cells were incubated with doxycycline to induce shRNA expression, and cell numbers were compared with parental non-transduced wildtype cells as well as to cells transduced with a non-targeting control shRNA (shctr). Data represent the mean values with SEM, n = 3. One-way-ANOVA testing with Bonferroni multi-comparison correction was performed. The significance is indicated by asterisks for the comparison of shctr versus sh2PAICS (***: p-value<0.001). PAICS protein levels were assessed via immunoblotting of cells with (Doxy +; shPAICS expressed) and without (Doxy -; no shPAICS expression) doxycycline treatment. Anti-Ezrin served as a loading control. ctr: cells transduced with control shRNA; kd: cells transduced with pTRIPZ sh2PAICS. (TIF) [file pone.0064873.s003.tif]

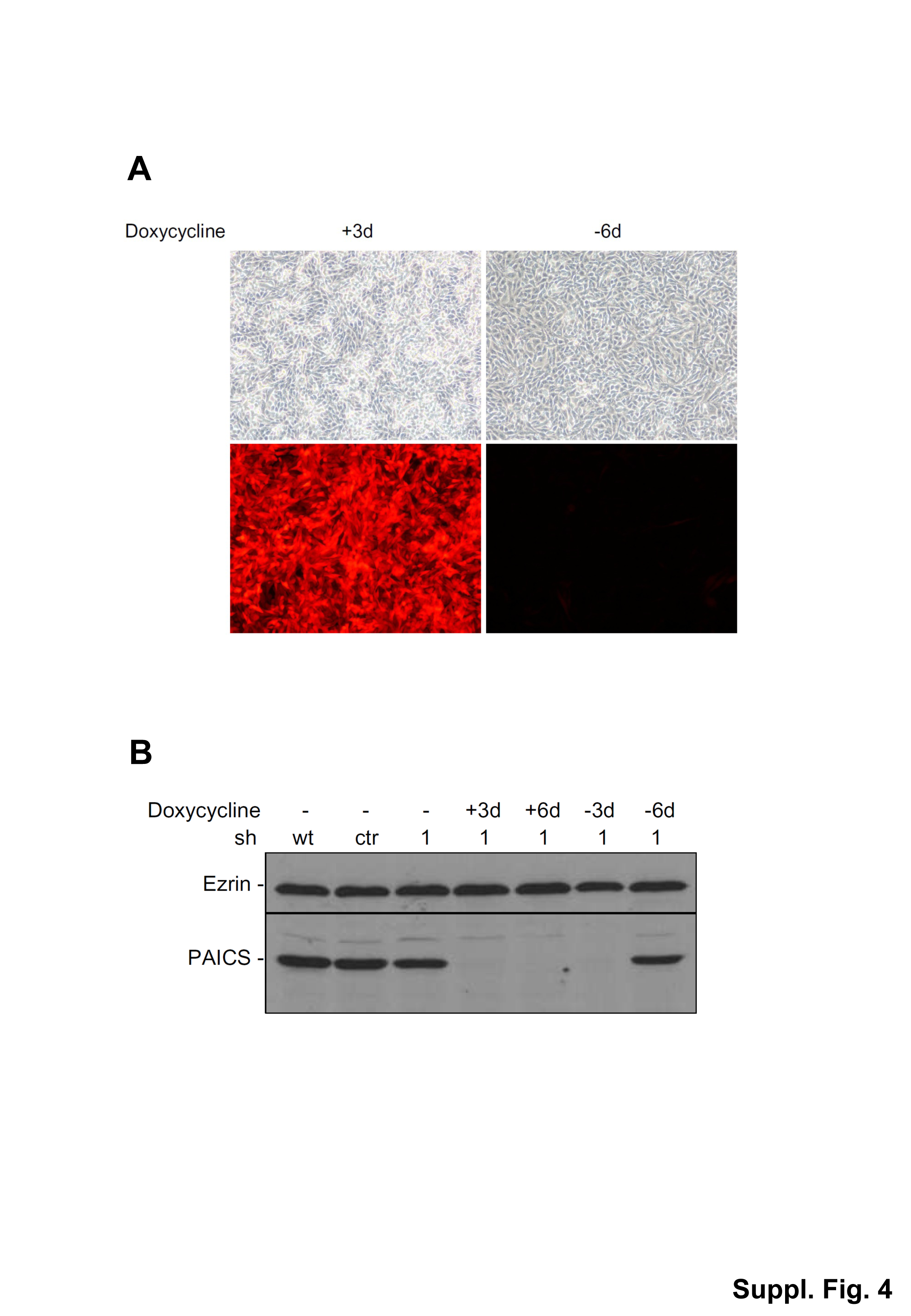

Supplement: Figure S4 — Inducible PAICS knockdown in MelJuSo cells. MelJuSo cells were transduced with the inducible shRNA vector pTRIPZ sh2PAICS. Doxycycline treatment induced the expression of both the turbo red fluorescent protein (tRFP) and PAICS-specific shRNA. A depicts images of pTRIPZ sh2PAICS-transduced MelJuSo cells after three days of doxycycline treatment (+3d) and images of the same cell culture after six days of doxycycline withdrawal (−6d). The upper panel represents transmitted-light images, while the lower panel displays red fluorescence images showing tRFP expression. B. A Western blot membrane incubated with anti-PAICS antiserum, which visualizes PAICS protein levels in cells without shRNA induction by doxycycline, and after both three and six days of incubation with 1 µg/ml doxycycline (induction of shRNA expression). Cell lysates prepared after three and six days of doxycycline withdrawal were also analyzed. Equal protein loading was confirmed by anti-EZRIN staining. (TIF) [file pone.0064873.s004.tif]

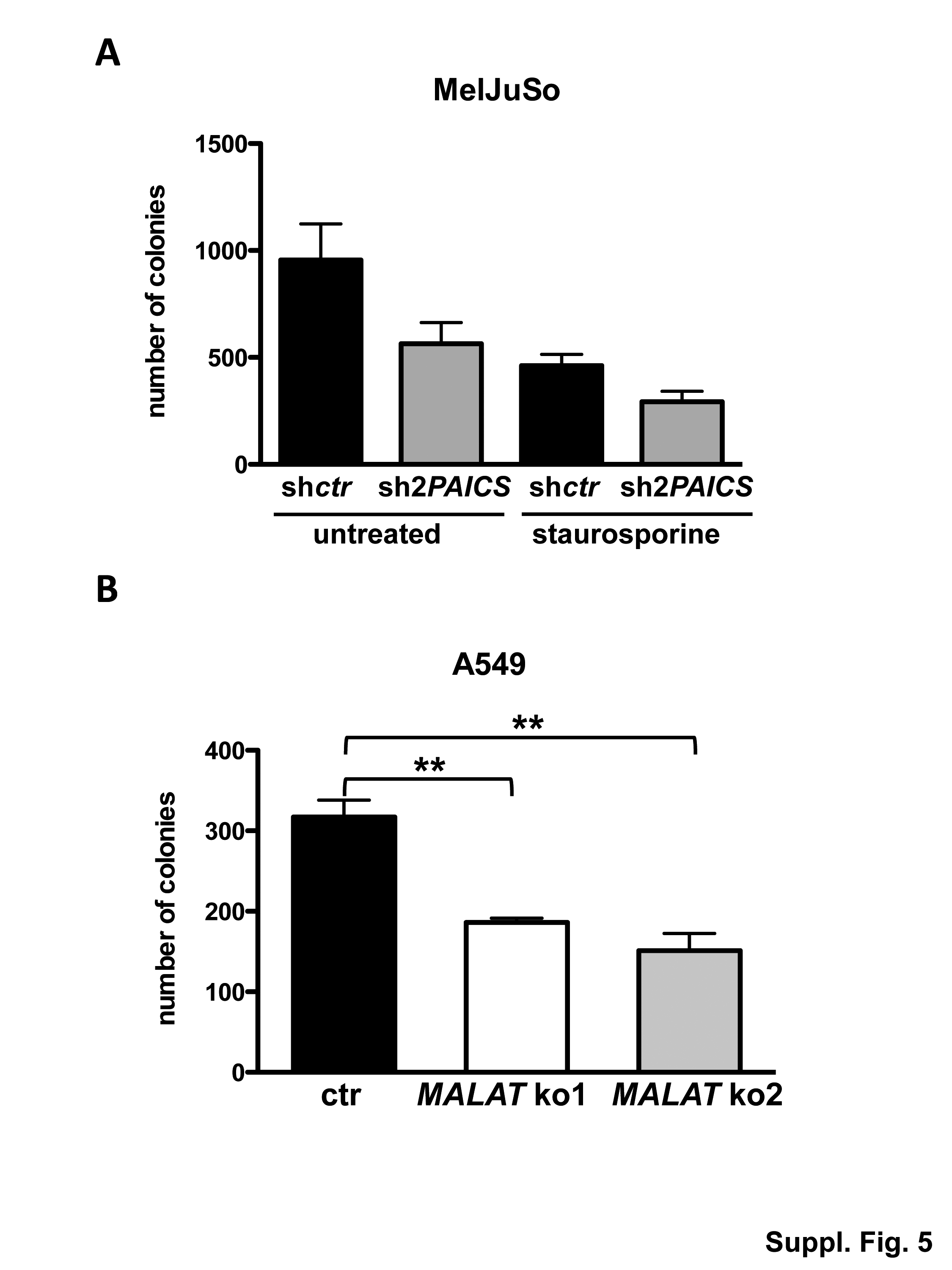

Supplement: Figure S5 — Colony formation assays with PAICS knockdown and MALAT1 knockout tumor cell lines. A. After pre-treatment for 3 days in medium with 1 µg/ml doxycycline, 2.5×10^3^ MelJuSo cells stably transduced with pTRIPZ sh2PAICS or shctr were seeded in methyl cellulose containing 40% RPMI medium (supplemented with 30% FCS, 3% penicillin/streptomycin and 3 µg/ml doxycycline). For the induction of apoptosis, the cells were incubated with 0.2 µM staurosporine 16 hours before seeding. Colony growth was quantified after 6 days. B. A549 cells (7.5x10^2^ cells per well) were plated in normal DMEM/10% FCS medium in triplicate in 6-well plates, and the colonies were allowed to grow for 8 days. GFP control cells were compared with the zinc-finger nuclease-mediated MALAT1 knockout clones, ko1 and ko2. The colonies were fixed and stained with crystal violet solution and counted in a 3×3 cm scoring grid. The experiments were performed three times, and the results are represented as mean ± SEM. *p<0.05; **p<0,01; one-way ANOVA analysis with Bonferroni multi-comparson correction. (TIF) [file pone.0064873.s005.tif]

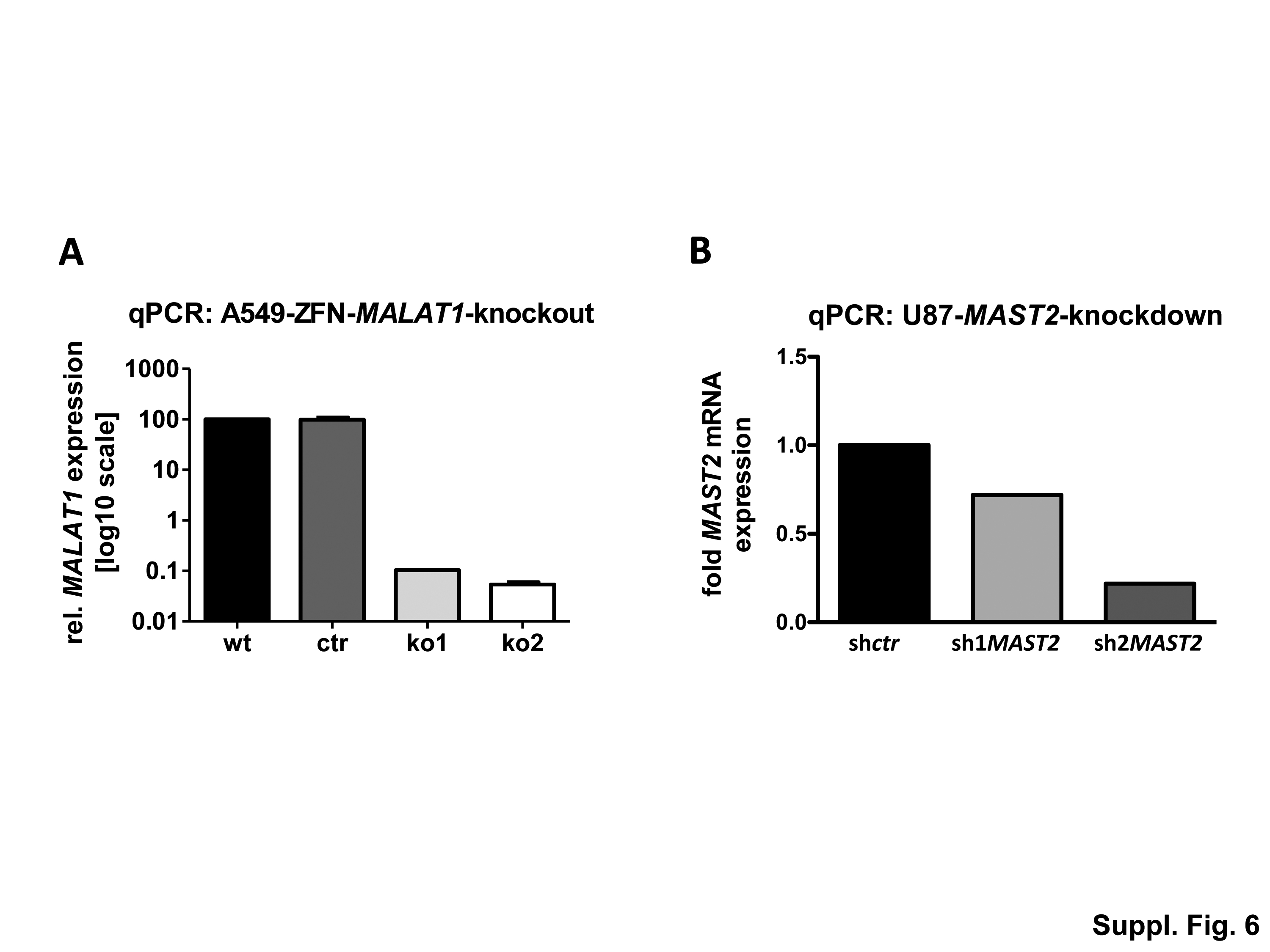

Supplement: Figure S6 — Quantitative PCR analysis of A549-ZFN- MALAT1 knockout and MAST2 shRNA knockdown U87 cells. A. The residual MALAT1 RNA levels expressed in A549-ZFN-MALAT1-knockout cells (ko1 and ko2) were compared with both control (ctr; cells with random GFP integration) and parental A549 (wt) cells by Real Time qPCR analysis. Data are presented as mean values ± SEM (n = 3) on a logarithmic scale (log10). B. The lentiviral MAST2 shRNA knockdown in U87 cells with two different MAST2 shRNA sequences was confirmed via qPCR in comparison with non-targeting control shRNA-transduced U87 cells. Relative MAST2 mRNA expression levels are presented from a single experiment. GAPDH and HPRT mRNA expression were used for relative quantification of MAST2 expression. (TIF) [file pone.0064873.s006.tif]

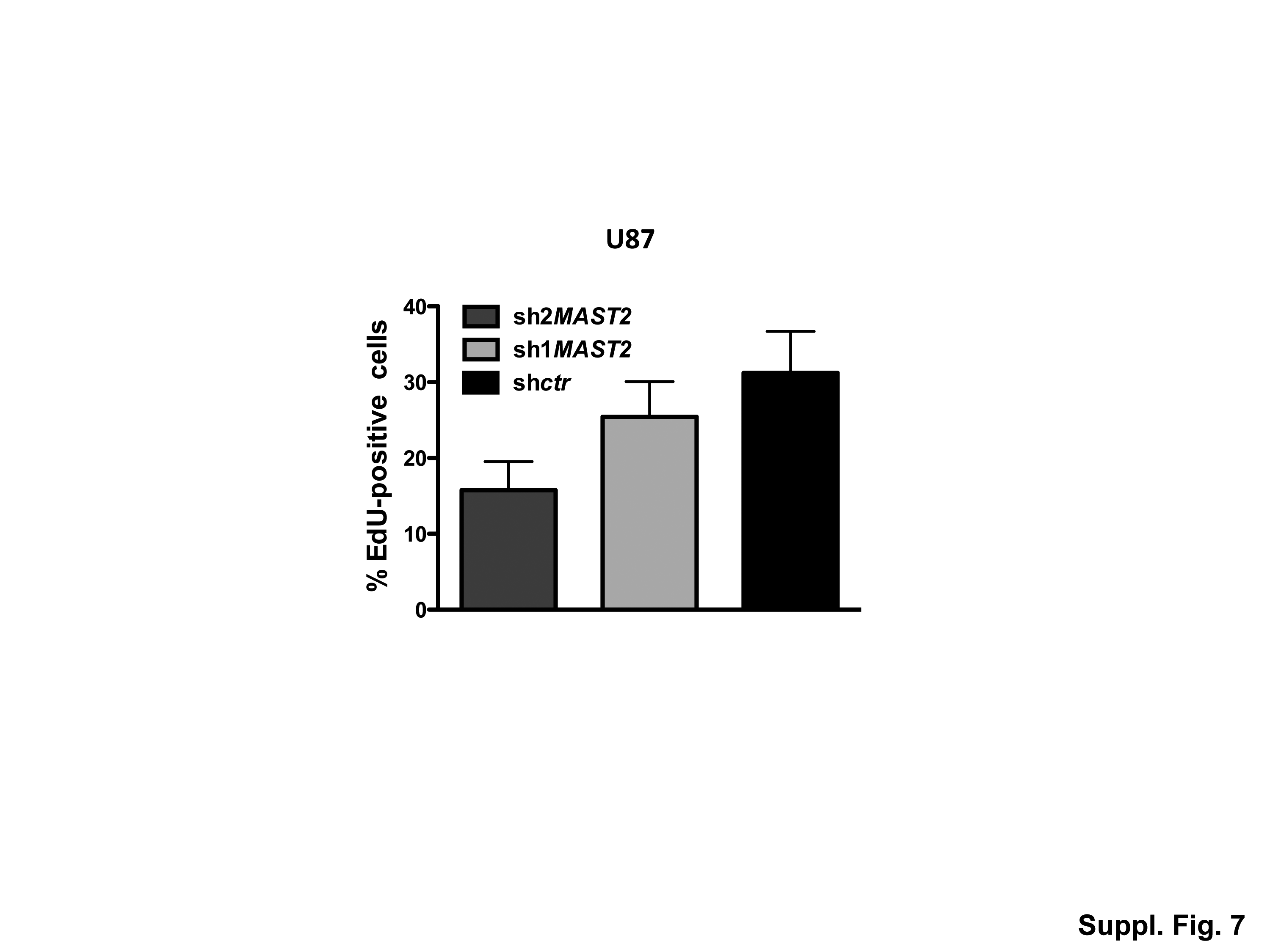

Supplement: Figure S7 — Quantification of proliferation upon shRNA-mediated knockdown of MAST2 . The two U87 MAST2 knockdown cell lines sh1MAST2 and sh2MAST2 were compared to control vector-transduced cells for their proliferation rate using the Click-iT® Edu Proliferation Assay kit (Alexa Fluor 488, Life Technologies). Cells were incubated with EdU for one hour, and the percentage of EdU-positive cells was quantified by FACS analysis. Data are presented as the mean values ± SEM (sh2MAST2: n = 3; sh1MAST2, shctr: n = 4). (TIF) [file pone.0064873.s007.tif]

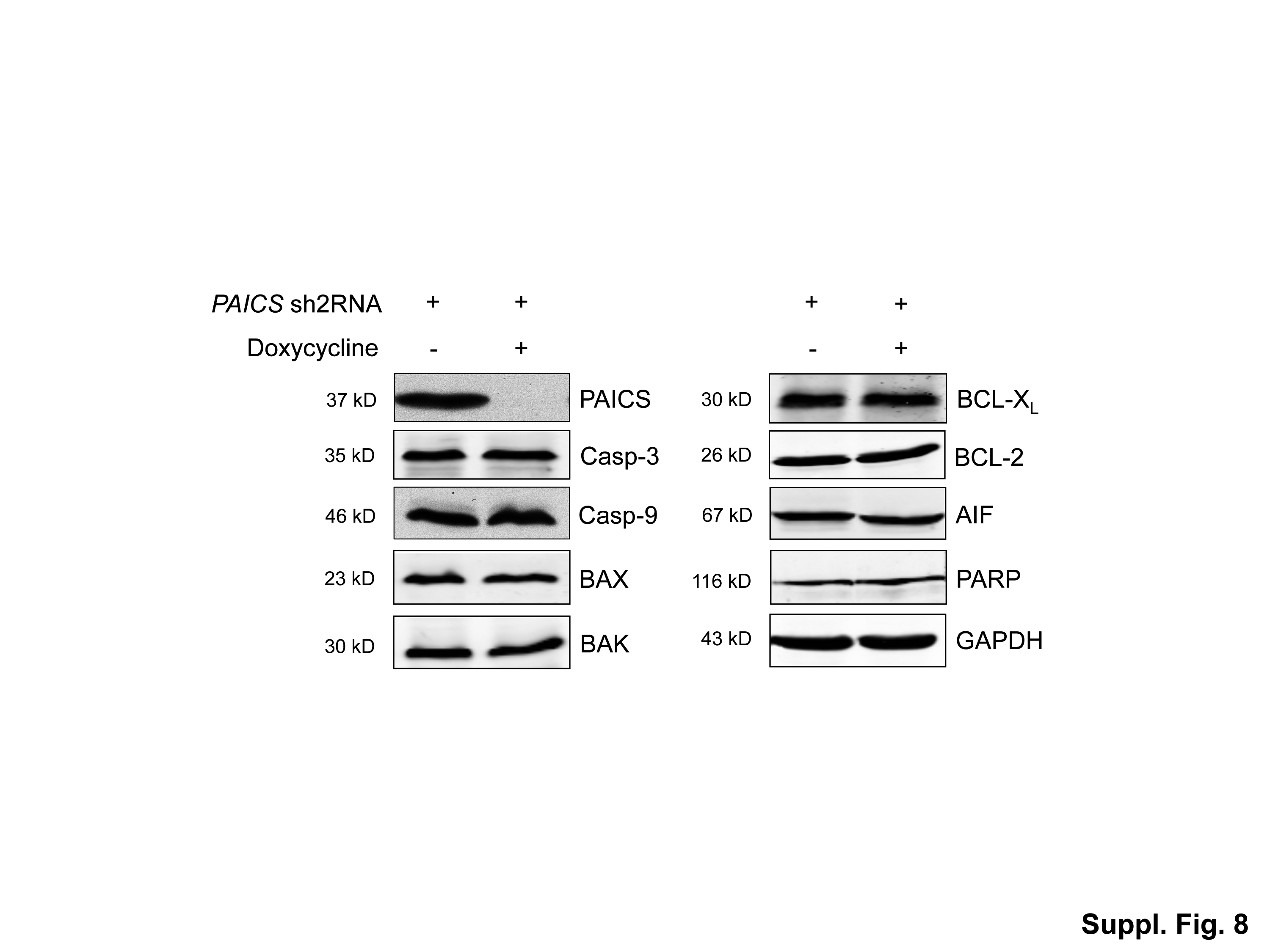

Supplement: Figure S8 — Expression levels of several apoptosis regulators in either the presence or absence of PAICS. The melanoma cell line, MelJuSo, was stably transduced with the lentiviral construct, pTRIPZ sh2PAICS, and the downregulation of PAICS was induced using doxycycline. 50 µg (per lane) of protein lysate was loaded to the gel and Western Blot analyses were performed either as described in the manuscript (for CASPASE-9) or using the Odyssey system (for all other proteins). The following antibodies were used: self-raised anti-PAICS antiserum (see manuscript), anti-CASPASE-3 (Cell Signaling, #9662), anti-CASPASE-9 (Alexis, #ALX-210-838-R100), anti-BAX (Upstate, #06-499), anti-BAK (Santa Cruz, #sc832, clone G-23), anti-BCL-xL (Cell Signaling, #2764, clone 54H6), anti-BCL-2 (Santa Cruz, #sc509, clone 100), anti-AIF (Chemicon, #AB16501), anti-PARP (Cell Signaling, #9542) and anti-GAPDH (Calbiochem, #CB1001). The following secondary antibodies were used with the Odyssey system: IRDye 680CW goat anti-rabbit (LI-COR, #926-32221) and IRDye 800CW goat anti-mouse (LI-COR, #926-32210). (TIF) [file pone.0064873.s008.tif]
