# Supplementary material for: A Functional Yeast Survival Screen of Tumor-Derived cDNA Libraries Designed to Identify Anti-Apoptotic Mammalian Oncogenes
Source: PLoS One. 2013 May 22;8(5):e64873. doi: 10.1371/journal.pone.0064873 (PMC3661464; doi:10.1371/journal.pone.0064873)
Supplement: Table S1 — Complete list of genes identified by functional yeast survival screening of cDNA libraries derived from a melanoma metastasis, leukemia samples and glioblastomas. (PDF) [file pone.0064873.s009.pdf]

Gene	NCBI Reference Sequence	Number of clones	Survival Screen
**ACSL4**	NM_022977		Mel/HC4
**ANXA1**	NM_000700		Mel/DSI
**ARGLU1**	NM_018011		Mel/HC4
**ARHGAP21**	NM_020824	2	Mel/DSI<br>Mel/HC4
**ARL5A**	NM_177985		Mel/DSI
**ATAD2**	NM_014109		Mel/HC4
**ATP1A2**	NM_000702		Glio/DSI
**ATP5E**	NM_001001977	2	Mel/HC4
**ATP5G3**	NM_001689	2	Mel/HC4<br>Leuk/HC4
**ATRX**	NM_138270		Mel/DSI
**BBX**	NM_001142568		Glio/DSI
**BCAS2**	NM_005872		Mel/DSI
**BDP1**	NM_018429		Leuk/HC4
**B2M**	NM_004048	21	Mel/DSI<br>Mel/HC4<br>Glio/DSI
**BMI1**	NM_005180		Mel/DSI
**BNIP3L**	NM_004331		Glio/DSI
**BPGM**	NM_001724		Mel/DSI
**CA1**	NM_001128829		Leu/HC4
**CALM2**	NM_001743	2	Mel/DSI
**CALU**	NM_001219		Mel/DSI
**CAPN2**	NM_001748		Mel/HC4
**CAPZA2**	NM_006136		Mel/DSI
**CBWD2**	NM_172003		Mel/DSI
**CCDC72**	NM_015933		Mel/HC4
**CCNC**	NM_005190		Mel/DSI
**CCNH**	NM_001239		Mel/DSI
**CCNL1**	NM_020307		Mel/HC4
**CDK6**	NM_001259	4	Leu/HC4
**CHMP5**	NM_016410		Mel/HC4
**CLIC4**	NM_013943		Mel/HC4
**CNIH**	NM_005776		Mel/HC4
**CNOT8**	NM_004779		Mel/DSI
**COL1A1**	NM_000088		Mel/DSI
**COL3A1**	NM_000090	3	Mel/DSI<br>Mel/HC4
**COL4A1**	NM_001845		Mel/DSI
**COPB1**	NM_016451		Mel/DSI
**COQ5**	NM_032314		Mel/HC4
**C7orf73**	NM_001130929		Mel/DSI
**C8orf4**	NM_020130		Mel/DSI
**C8orf40**	NM_138436		Mel/HC4
**C8orf46**	NM_152765		Glio/DSI
**C11orf58**	NM_014267		Mel/DSI
**CTGF**	NM_001901		Mel/DSI
**CTNNB1**	NM_001904		Mel/HC4
**CXCL10**	NM_001565		Glio/DSI
**CYBB**	NM_000397		Glio/DSI
**CYR61**	NM_001554		Mel/DSI
**DAD1**	NM_001344		Mel/HC4
**DC2**	NM_021227	2	Mel/HC4
**DCN**	NM_001920	3	Mel/HC4<br>Glio/DSI
**DPYD**	NM_000110		Mel/DSI
**EFEMP1**	NM_001039348		Mel/HC4
**EIF4A2**	NM_001967	5	Mel/DSI
**EIF4B**	NM_001417		Mel/HC4
**EIF4E**	NM_001968		Glio/DSI
**EIF3S10**	NM_003750		Mel/DSI
**EPC2**	NM_015630		Mel/DSI
**EPS15**	NM_001981		Mel/HC4
**ERO1L**	NM_014584		Mel/HC4
**FAM36A**	NM_198076		Glio/DSI
**FAM107B**	NM_031453		Mel/HC4
**FBXO8**	NM_012180		Mel/HC4
**FLT3**	NM_004119		Leu/HC4
**FSTL1**	NM_007085		Mel/DSI
**FUNDC1**	NM_173794		Mel/DSI
**FYTTD1**	NM_032288		Mel/HC4
**GABARAPL2**	NM_007285		Mel/DSI
**GAS1**	NM_002048		Glio/DSI
**GBAS**	NM_001483		Mel/DSI
**GLUL**	NM_002065		Glio/DSI
**GNAS**	NM_000516	3	Mel/HC4
**GOLT1B**	NM_016072		Mel/DSI
**GPM6A**	NM_201592	2	Glio/DSI
**GTDC1**	NM_001006636		Mel/DSI
**HBLD2**	NM_030940		Mel/DSI
**HEG1**	NM_020733		Mel/HC4
**H3F3B**	NM_005324		Mel/HC4
**HIGD1A**	NM_014056	4	Mel/DSI<br>Mel/HC4<br>Glio/DSI
**HMGB1**	NM_002128		Mel/HC4
**HMGN3**	NM_004242	2	Mel/HC4
**HNRF**	NM_001098208		Glio/DSI
**HNRNPA1**	NM_031157		Mel/DSI
**IFIT1**	NM_001548		Glio/DSI
**IGFBP3**	NM_001013398	2	Mel/DSI
**IGJ**	NM_144646		Mel/HC4
**JMJD1C**	NM_004241		Mel/HC4
**LACTB2**	NM_016027		Glio/DSI
**LAP3**	NM_015907		Mel/DSI
**LBH**	NM_030915		Mel/DSI
**LIFRAS**	BC038371		Glio/DSI
**LONRF1**	NM_152271		Glio/DSI
**LRIF1**	NM_001006945		Glio/DSI
**LTBP1**	NM_000627		Glio/DSI
**LUM**	NM_002345	2	Mel/DSI
**LYPLA1**	NM_006330		Glio/DSI
**MALAT1**	NR_002819	11	Mel/HC4<br>Leuk/HC4
**MAP7D3**	NM_024597		Mel/DSI
**MAST2**	NM_015112		Glio/DSI
**MBD2**	NM_003927	2	Mel/DSI
**MCP-1**	NM_002982		Glio/DSI
**MGST1**	NM_020300	2	Mel/HC4<br>Glio/DSI
**MIER3**	NM_152622		Glio/DSI
**MKI67IP**	NM_032390	2	Mel/DSI<br>Glio/DSI
**MORF4L2**	NM_012286	3	Mel/HC4<br>Mel/DSI<br>Glio/DSI

**MPHOSPH6**	NM_005792		Mel/DSI
**MRPL13**	NM_014078		Glio/DSI
**MRPL39**	NM_017446		Mel/DSI
**NAMPT**	NM_005746		Glio/DSI
**NAP1L1**	NM_139207		Mel/HC4
**NARG2**	NM_024611		Mel/HC4
**NDUFB5**	NM_002492	2	Glio/DSI
**NDUFC1**	NM_002494		Mel/HC4
**NFIA**	NM_005595		Mel/DSI
**NR2C2**	NM_003298		Mel/HC4
**NR3C1**	NM_000176	2	Leu/HC4
**NT5C3**	NM_001002010		Mel/HC4
**NUDT5**	NM_014142		Mel/HC4
**NUDT21**	NM_007006	2	Mel/DSI
**NUP107**	NM_020401		Mel/DSI
**OAF**	NM_178507		Mel/HC4
**OAT**	NM_000274	2	Mel/HC4
**OMG**	NM_002544		Glio/DSI
**PAICS**	NM_006452	4	Mel/DSI<br>Mel/HC4
**PDK4**	NM_002612		Mel/HC4
**PDZD8**	NM_173791		Mel/DSI
**PGK1**	NM_000291		Leu/HC4
**PGRMC1**	NM_006667	2	Mel/HC4
**PJA2**	NM_014819		Glio/DSI
**PLSCR1**	NM_021105		Mel/HC4
**PMP2**	NM_002677	2	Glio/DSI
**PMP22**	NM_000304		Mel/DSI
**PMS1**	NM_000534		Mel/HC4
**POLR3A**	NM_007055		Leuk/HC4
**PPA1**	NM_021129		Mel/DSI
**PPAP2B**	NM_003713		Mel/DSI
**PPP1CC**	NM_002710		Mel/DSI
**PPP2CA**	NM_002715		Mel/HC4
**PRDX3**	NM_006793		Glio/DSI
**PRNP**	NM_183079		Mel/HC4
**PRRC1**	NM_130809		Mel/HC4
**PRSS23**	NM_007173	2	Mel/DSI
**PSAT1**	NM_058179		Glio/DSI
**PTPLAD1**	NM_016395		Mel/DSI
**RAB11A**	NM_004663	2	Leu/HC4
**RAP1GDS1**	NM_021159		Mel/HC4
**RAP2A**	NM_021033		Mel/DSI
**RCN2**	NM_002902	3	Glio/DSI
**REXO2**	NM_015523	3	Mel/HC4
**RGS2**	NM_002923		Mel/HC4
**RGS5**	NM_003617		Glio/DSI
**RHOA**	NM_001664		Mel/DSI
**RIOK2**	NM_018343		Glio/DSI
**RNPC3**	NM_017619		Leuk/HC4
**RPL4**	NM_000968		Mel/HC4
**RPL15**	NM_002948		Glio/DSI
**RPL23a**	NM_000984	2	Leu/HC4
**SBDS**	NM_016038		Mel/DSI
**SCAMP1**	NM_004866		Mel/DSI
**SCML1**	NM_001037536		Mel/DSI
**SC4MOL**	NM_006745		Mel/DSI
**SCYE1**	NM_004757		Glio/DSI
**SDHD**	NM_003002	2	Mel/DSI<br>Glio/DSI
**SEC24D**	NM_014822	2	Mel/DSI
**SELT**	NM_016275		Glio/DSI
**SFRP2**	NM_003013		Mel/DSI
**SFRS11**	NM_004768		Glio/DSI
**SGK1**	NM_005627		Glio/DSI
**SLC38A2**	NM_018976	4	Mel/DSI<br>Leuk/HC4
**SLC39A6**	NM_012319		Mel/HC4
**SNAP25**	NM_130811		Glio/DSI
**SNRPB2**	NM_198220		Glio/DSI
**SNX6**	NM_152233		Mel/HC4
**SPARCL1**	NM_004684		Glio/DSI
**SPCS2**	NM_014752	4	Mel/DSI<br>Leuk/HC4
**SPP1**	NM_001251830	6	Glio/DSI
**SPPL3**	NM_139015		Leuk/HC4
**SPRED2**	NM_181784		Glio/DSI
**SPTBN1**	NM_003128		Mel/HC4
**SRP14**	NM_003134		Leu/HC4
**SSR1**	NM_003144	2	Mel/DSI<br>Leuk/HC4
**STAM2**	NM_005843		Mel/HC4
**STEAP1**	NM_012449		Mel/HC4
**SUCLA2**	NM_003850		Mel/HC4
**TAF2**	NM_003184		Mel/HC4
**TBC1D7**	NM_016495		Glio/DSI
**TGFBR2**	NM_003242		Mel/DSI
**THUMPD1**	NM_017736		Mel/DSI
**TLOC1**	NM_003262		Mel/HC4
**TMEM50A**	NM_014313		Mel/HC4
**TMEM66**	NM_016127	2	Mel/DSI<br>Glio/DSI
**TMEM165**	NM_018475		Glio/DSI
**UBE2D1**	NM_003338		Mel/HC4
**UBE2V1**	NM_001032288	2	Mel/DSI<br>Mel/HC4
**UBE2V2**	NM_003350	2	Mel/HC4<br>Mel/DSI
**UFM1**	NM_016617		Mel/HC4
**VDP**	NM_003715		Mel/DSI
**VPS29**	NM_016226	2	Mel/DSI<br>Glio/DSI
**VPS37A**	NM_152415		Mel/HC4
**WDR68**	NM_005828		Mel/DSI
**ZCCHC10**	NM_017665		Mel/DSI
**ZCCHC11**	NM_001009881		Glio/DSI
**ZDHHC17**	NM_015336		Mel/HC4
**ZNF403**	NM_024835		Mel/HC4
**ZSWIM6**	NM_020928		Mel/HC4

Suppl. Table 1
